# Outer membrane proteins mediate unconventional secretion of *Pseudomonas* peroxidase through crosstalk between the Sec pathway and outer membrane vesicles

**DOI:** 10.1371/journal.pgen.1012311

**Published:** 2026-09-15

**Authors:** Congying Liang, Wenping Zhu, Lu Lin

**Affiliations:** 1 Institute of Marine Science and Technology, Shandong University, Qingdao, China; 2 Southern Marine Science and Engineering Guangdong Laboratory (Zhuhai), Zhuhai, China; 3 Shandong Key Laboratory of Intelligent Marine Engineering Geology, Environment and Equipment, Qingdao, China; Tufts University School of Medicine, UNITED STATES OF AMERICA

## Abstract

Lipoprotein and OmpW are two major components of the outer membrane (OM) in Gram-negative bacteria and play essential roles in various physiological processes, e.g., protein secretion, folding, and localization. They typically assist in maintaining client proteins in a folded state or form pore channels during secretion. However, how OM proteins facilitate the secretion of proteins that lack classical signal peptides remains elusive. Here, we demonstrate that they contribute to the secretion of unconventional B-type dye-decolorizing peroxidase (DypB_2985_) in *Pseudomonas putida*. The lipoprotein, Lpp_1528_, which contains a Sec signal peptide, is translocated to the periplasm through the Sec pathway and anchored in the inner leaflet of the OM. Lpp_1528_ recognizes the C-terminal hydrophobic region of DypB_2985_ in the cytoplasm and facilitates its coupling to the Sec machinery for inner membrane translocation, despite DypB_2985_ lacking a canonical N-terminal Sec signal peptide. Following translocation, the two proteins appear to dissociate in the periplasm. Subsequently, another OM protein, OmpW_4836_, recognizes the N-terminal hydrophobic region of periplasmic DypB_2985_ and mediates its incorporation into outer membrane vesicles (OMVs) for extracellular delivery. Our study reveals the crosstalk between the Sec pathway and OMVs in the secretion of a non-canonical peroxidase, in which OM proteins, acting as the molecular tethers, mediate the stepwise secretion process. It expands our understanding of non-classical protein secretion mechanisms and bacterial survival strategies. Moreover, the identified OMV sorting mechanism offers potential for the further functionalization of OMVs as versatile biotechnological platforms.

## Author summary

Bacterial protein secretion is a fundamental physiological process. Proteins commonly contain signal peptides and are exported via classical secretory pathways. However, some proteins without any characterized signal sequences can also leave the cytoplasm and be transported across cell membranes, posing a major unresolved question in the field. Here, we provide insights into the secretion of unconventional B-type dye-decolorizing peroxidases (DypB_2985_) in *Pseudomonas putida*. The lipoprotein, Lpp_1528_, identifies DypB_2985_ in the cytoplasm and targets it to the Sec secretion components, which transport DypB_2985_ across the inner membrane. Subsequently, another outer membrane protein, OmpW_4836_, recognizes periplasmic DypB_2985_ and mediates its sorting into OMVs for extracellular delivery. Overall, this study advances the understanding of non-classical protein secretion pathways and the inclusion sorting mechanism of OMVs, raising the possibility for the development of tailored biocatalytic and delivery platforms.

## Introduction

Protein transport is not only an important physiological process in bacterial systems, but also acts as a communication tool between an organism and its surrounding environment for signal transduction, nutrient acquisition and other vital activities [1,2]. Exported proteins commonly contain known signal peptides or secretion motifs. They are synthesized as precursors in cytoplasm and are then exported through multiple classical secretion pathways, including the general secretion pathway (Sec pathway), the signal recognition particle pathway (SRP pathway), and the twin arginine translocation pathway (Tat pathway) [3–5]. The Sec pathway is ubiquitous and essential for bacterial viability. SecB, a molecular chaperone of the Sec pathway, recognizes the preprotein via the N-terminal Sec signal peptide. It maintains the preprotein in an unfolded conformation, and delivers it to the motor ATPase SecA, which subsequently drives the preprotein across the SecYEG protein channel in the cytoplasmic membrane [6]. The SRP pathway specifically recognizes the hydrophobic N-terminal signal peptide emerging from a nascent polypeptide by the signal recognition particle (SRP). The ribosome nascent chain complex is then threaded through the SecYEG translocon [7]. In contrast, TatC in the Tat pathway recognizes the N-terminal Tat signal sequence of a fully folded protein and targets it to the membrane-embedded TatABC complex for translocation across the cytoplasmic membrane [8].

A growing number of proteins without any classical signal peptides or secretion motifs have been found to be secreted into the surrounding environment. We recently reported a secretion model for an unconventional B-type dye-decolorizing peroxidase (DypB_3152_) in *Pseudomonas putida* A514 [9]. DypB_3152_ interacts with the Type VI secretion system (T6SS) components (VgrG and Hcp) for periplasmic delivery. Next, OMVs package the enzyme for extracellular release. This study demonstrates that bacteria can crosslink existing systems for the secretion of proteins that lack conventional secretion signals. Another prominent strategy for non-classical secretion involves the utilization of an accessory protein that links the cognate effector to a component of the secretion pathway. One well-known adaptor protein is SecA2 in Gram-positive bacteria, which enables Sec-dependent export of cargos lacking canonical Sec signal peptides [10]. For instance, the preprotein of *Listeria monocytogenes* MnSod lacks a typical Sec signal peptide and instead relies on SecA2 to recognize a N-terminal hydrophobic patch, enabling its export through the Sec pathway [11]. The terminal sequences of non-classically secreted proteins show low similarity in either amino acid sequences or characteristic features, in contrast to the conserved signal peptides or motifs found in canonical secreted proteins [12]. This variability suggests that non-classical secretion mechanisms are highly diverse and likely tailored to individual substrates. One example is provided by the two ligninolytic DypBs in *P. putida* A514, DypB_3152_ and DypB_2985_ [13]. Both proteins are detected in the periplasmic and extracellular spaces, yet they lack any conventional N- or C-terminal signal sequences [9,13,14]. A crosslink between T6SS and OMV translocation is responsible for the delivery of DypB_3152_, but not for DypB_2985_. Undoubtedly, it is a challenge to uncover the secretion mechanisms of individual non-classically secreted proteins.

To untangle the secretion processes of unconventionally secreted proteins, two essential questions should be addressed. One is the recognition strategy. How does a transporter distinguish the secreted proteins from all proteins in the cytoplasm? Proteins with classical signal peptides, which rely on the Sec, Tat or SRP pathways, are recognized by secretory components through N-terminal signal sequences. Similarly, non-classically secreted proteins may harbor short hydrophobic motifs at the N- or C-terminus, which can be recognized by accessory proteins, even when not cleaved after secretion [12]. SecA2 in Sec pathway and DUF4123 in T6SS are both well-known adaptor-like proteins to target cargo proteins without N-terminal conserved domains, providing a connection between secretory components and cargos [15–17]. However, the low number of currently known accessory proteins contrasts sharply with the growing number of non-canonically secreted proteins. Moreover, it remains an open question of how secretory components recognize a diverse repertoire of unconventional exported proteins. The second point is the translocation process, which involves the secretion channels spanning cell membranes (e.g., inner and outer cell membranes). SecYEG in the Sec pathway and TatABC in the Tat pathway are classical protein complexes embedded in the cytoplasmic membrane that form translocation channels [2]. In addition, OMVs, which bud and pinch off from the OM, act as vital delivery vehicles to export cytoplasmic and periplasmic proteins to the extracellular environment, especially in Gram-negative bacteria. It is inferred that the enriched cargos in OMVs should be determined by some uncharacterized sorting mechanism, instead of random packaging [18]. However, the mechanisms by which bacteria selectively sort and package specific cargo proteins into OMVs remain poorly understood, particularly for non-classically secreted proteins that lack signal peptides or conserved sorting motifs.

In this study, we employed *Pseudomonas* DypB_2985_ as the target to explore the secretory pathways of non-classically secreted proteins. Co-immunoprecipitation coupled with mass spectrometry (co-IP-MS) analysis identified candidate proteins involved in DypB_2985_ secretion as outer membrane proteins. Subsequent gene deletion and protein level analyses pinpointed that the lipoprotein Lpp_1528_ facilitates translocation across the inner membrane, while the outer membrane protein OmpW_4836_ mediates the export of DypB_2985_ into OMVs for extracellular delivery. Lpp_1528_ contains a canonical Sec signal sequence and interacts with components of the Sec pathway, including SecA, SecB and SecYEG, thereby enabling its secretion via the Sec pathway. During this process, Lpp_1528_ recognizes the C-terminal hydrophobic region of DypB_2985_ in the cytoplasm and recruits the Sec signal peptide-deficient DypB_2985_ to the Sec machinery for translocation across the inner membrane. After entering the periplasm, the two transiently interacting proteins appear to dissociate. DypB_2985_ is then recognized by OmpW_4836_ via the N-terminal hydrophobic region and packaged into OMVs for extracellular release. This study elucidates the export mechanism of the non-canonical DypB_2985_ and expands the repertoire of accessory proteins involved in non-classical secretion. Moreover, it sheds light on the mechanism of protein sorting for OMV incorporating, facilitating the development of customized OMV-based biotechnological platforms.

## Results

### Co-IP-MS reveals the candidates involved in the secretion of unconventional DypB_2985_

**Fig 1:**
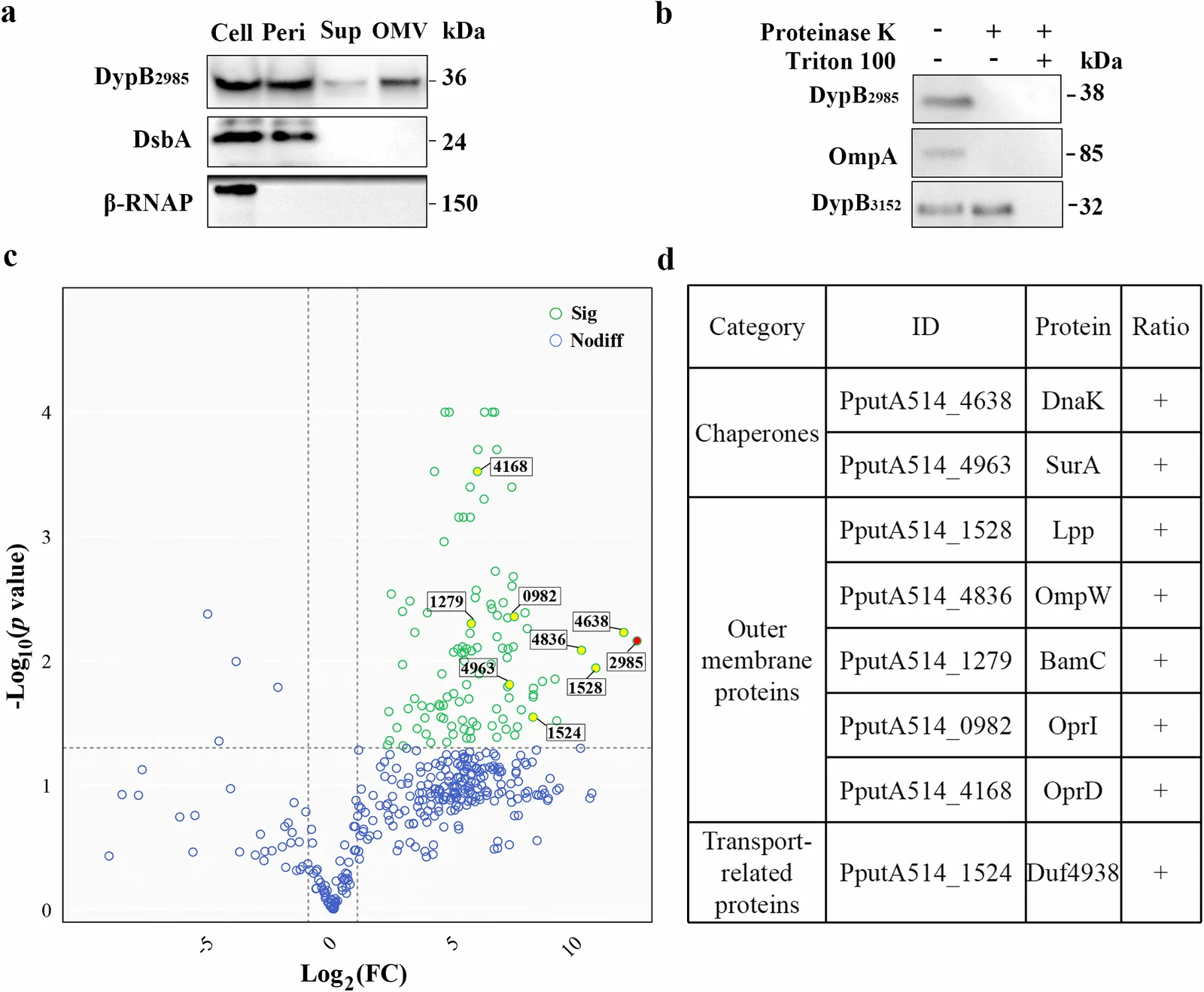
Extracellular secretion of DypB_2985_ and its associated putative transport proteins. **(a)** Western blot analysis of DypB_2985_ localization in cellular lysate (Cell), periplasmic fraction (Peri), culture supernatant (Sup), and OMVs from A_2985_, using an anti-His antibody. Cytoplasmic β-RNA polymerase (β-RNAP) and periplasmic disulfide bond-forming protein A (DsbA) were used as loading controls for total cellular lysate and periplasmic protein levels, respectively. **(b)** Subcellular localization of DypB_2985_ in OMVs, detected with an anti-flag antibody. OmpA was used as a reference for OMV surface proteins, while DypB_3152_ served as a control for OMV-resident proteins. Data from (a-b) are representative of two independent biological replicates. **(c)** Volcano plot of co-IP-MS data. The IgG group was used as a negative control. Significantly enriched proteins (Sig) are defined as those with log_2_ (FC) > 1 and *p* < 0.05, where FC represents fold change in protein abundance between the Flag-DypB_2985_ group and IgG control group. DypB_2985_ is highlighted in red. Eight proteins previously implicated in molecular translocation are marked in yellow. Data were derived from three biological replicates and analyzed using a two-sided Student’s *t*-tes*t*. **(d)** List of transport-related proteins enriched in the Flag-DypB_2985_ group. “+”: Significant enrichment of the corresponding protein in the Flag-DypB_2985_ sample compared to the IgG control.

DypB_2985_ is an Mn^2+^ independent lignin oxidizing enzyme [13], previously observed in the extracellular secretome of *P. putida* A514 [14]. Here, western blot analysis revealed the levels of DypB_2985_ in the periplasmic and extracellular compartments (including OMVs and culture supernatant) of A_2985_, in which A514 carries a copy of *dypB*_*2985*_ gene with a N-terminal His-tag on the pUCP_min_ plasmid (Fig 1a and Table A in S1 Appendix). It is well established that *P. putida* A514 produces OMVs packaging DypB_3152_, an OMV-resident protein, into the extracellular milieu [9]. The absence of the membrane protein SecY [19] in OMV fractions, coupled with the lack of detectable DypB_3152_ in total membrane extracts, confirms the purity of the isolated OMVs and rules out cross-contamination between the two fractions (Fig A in S1 Appendix). In this study, DypB_2985_ was also detected in OMV preparations (Fig 1a), suggesting its involvement in OMV-mediated secretion. Treatment of OMVs with proteinase K led to the degradation of DypB_2985_, exhibiting protease sensitivity comparable to that of outer membrane protein A (OmpA), a canonical OMV surface marker [20]. In contrast, DypB_3152_, a protein known to be encapsulated within OMVs [9], remained intact (Fig 1b). These results support the surface localization of DypB_2985_ on OMVs. Together, these data demonstrate that DypB_2985_ is likely to be secreted to the extracellular milieu via OMVs, with periplasmic transit preceding its incorporation into OMVs. However, signal peptide prediction tools failed to identify any conventional N- or C-terminal signal sequences in DypB_2985_, e.g., SignalP 6.0, TargetP 2.0 and SecretomeP 2.0 [21–23]. This indicates that DypB_2985_ secretion is likely not directly dependent on canonical secretion pathways, e.g., Sec and Tat pathways, which typically require recognition of signal peptides [2,24].

To uncover the non-canonical secretion mechanism of DypB_2985_, co-IP-MS was first employed to investigate the candidate proteins that might interact with DypB_2985_. A total of 109 proteins were significantly enriched in the Flag-DypB_2985_ group, compared to the IgG control group (Fig 1c and Table B in S1 Appendix). Among these, eight proteins had previously been implicated in molecular translocation and could be grouped into three functional categories. i) The first cluster consisted of chaperones, e.g., DnaK_4638_ and SurA_4963_. DnaK is a classical chaperone known for its role in protein folding [25], and has also been reported to facilitate the translocation of unfolded proteins across the cytoplasmic membrane by preventing their aggregation and maintaining them in a translocation-competent state [26,27]. In contrast, SurA is an important chaperon involved in the biogenesis of OM proteins. It prevents the aggregation of unfolded OM proteins and facilitates their delivery to the BAM complex that is embedded in the OM [28]. ii) The second cluster included lipoprotein and OMV associated proteins, e.g., Lpp_1528_, OmpW_4836_, OprD_1944_, OprI_0982_, and BamC_1279_ [29–31]. One lipoprotein, LppX, has been reported to function as a molecular chaperone that ensures the proper folding of xylanase during transport across the inner membrane [32]. Among the OMV associated proteins, OmpW has been specifically implicated in the transport of small hydrophobic molecules, including alkanes and metal ions [33]. iii) The third cluster contained transport-related proteins, exemplified by the protein with a domain of unknown function (DUF4938_1524_). DUF proteins, with unknown functional domains, are closely associated with protein secretion [34,35]. Based on these findings, the top four most abundant proteins, DnaK_4638_, Lpp_1528_, OmpW_4836_, and DUF4938_1524_, were selected as a refined subset (Fig 1d). These proteins represented all three functional categories, showed correlations with molecular transport, and were successfully overexpressed in the A514 strain.

**Fig 2:**
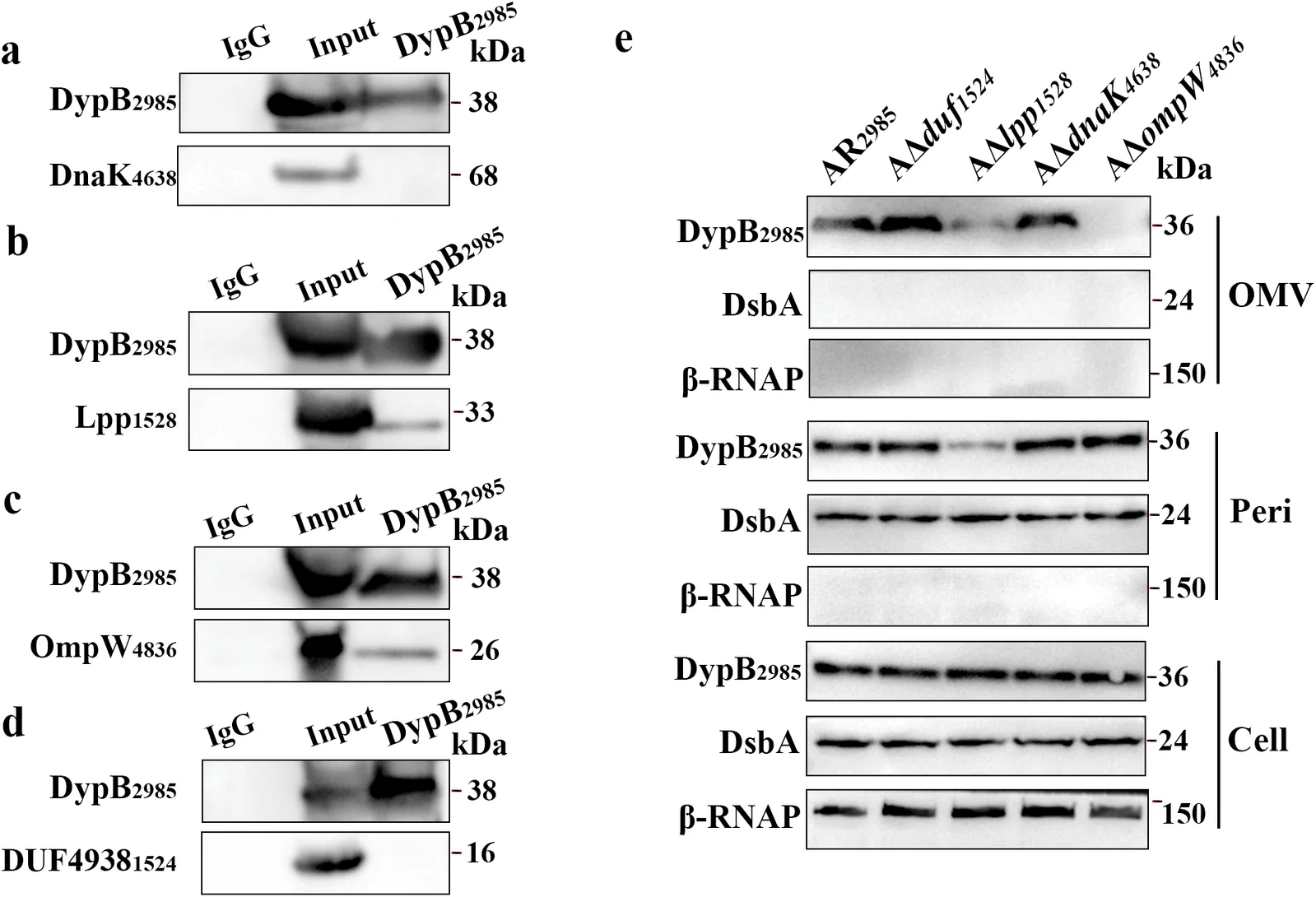
Putative transport proteins associated with DypB_2985_ secretion. (a-d) co-IP assay assessing the interaction between DypB_2985_ and the putative transport proteins DnaK_4638_ **(a)**, Lpp_1528_ **(b)**, OmpW_4836_ **(c)**, and DUF4938_1524_ **(d)**. The input cellular lysates and proteins co-precipitated with DypB_2985_ were detected by Western blot using anti-Flag and anti-Myc antibodies, respectively. An IgG control group was included to exclude non-specific binding to antibodies or magnetic beads. Data shown are representative of two independent biological replicates. **(e)** Western blot analysis of DypB_2985_ protein levels in cellular lysates (Cell), periplasmic (Peri), and OMVs of the relevant mutants, detected using an anti-His antibody. AR_2985_ was used as the blank reference. Data are representative of three biological replicates.

Co-immunoprecipitation (co-IP) assays were conducted to validate their interactions with DypB_2985_. Two of them, Lpp_1528_ and OmpW_4836_, showed interactions with DypB_2985_ (Fig 2a-2d). Moreover, the corresponding four genes were individually deleted in the AR_2985_ strain, generating the knockout mutants A∆*dnaK*_*4638*_, A∆*ompW*_*4836*_, A∆*lpp*_*1528*_, and A∆*duf4938*_*1524*_, respectively (Table A in S1 Appendix). AR_2985_, which harbored the genome editing elements Cas9n and λ-Red in the A_2985_ chromosome [36], exhibited DypB_2985_ protein levels comparable to those in the parental A_2985_ strain (Fig B in S1 Appendix), and was therefore used as the host strain for investigation DypB_2985_ secretion. Western blot analysis showed that deletion of *lpp*_*1528*_ led to a significant 60% reduction in the periplasmic DypB_2985_ level, but did not impair the intracellular DypB_2985_ protein level (Figs 2e and C(a) in S1 Appendix). Correspondingly, periplasmic DypB_2985_ activity in A∆*lpp*_*1528*_ was also significantly reduced by 53% (Fig C(b) in S1 Appendix). In addition, A∆*ompW*_*4836*_ completely abolished extracellular DypB_2985_ level in OMVs, but did not affect the abundance or enzymatic activity of periplasmic DypB_2985_ (Figs 2e, C and D in S1 Appendix). In contrast, A∆*dnaK*_*4638*_ and A∆*duf4938*_*1524*_ did not affect the intracellular or extracellular levels of DypB_2985_ compared to AR_2985_, confirming that they are not directly involved in the secretion of DypB_2985_ (Figs 2e, C and D in S1 Appendix). Together, these results indicate that Lpp_1528_ and OmpW_4836_ play a role in the transmembrane translocation of DypB_2985_.

### C-terminal residues of DypB_2985_ are required for interaction with Lpp_1528_

**Fig 3:**
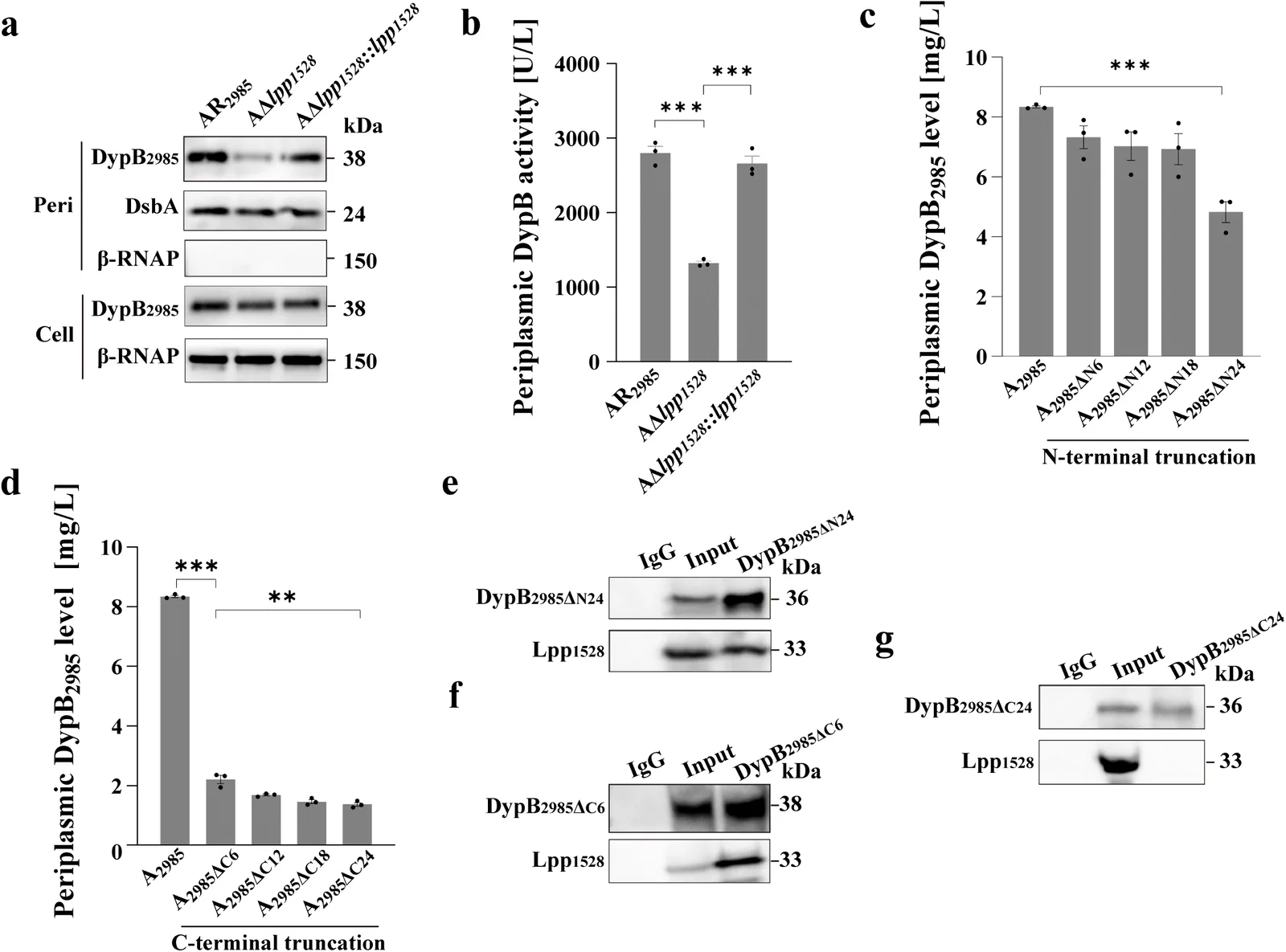
Lpp_1528_ mediates DypB_2985_ translocation across the inner membrane. (a) Western blot analysis of DypB_2985_ levels in cellular lysate (Cell) and periplasmic space (Peri) of the relevant mutants, probed with an anti-His antibody. β-RNAP and DsbA proteins were used as loading controls. AR_2985_ was the blank control strain. Data are representative of three biological replicates. (b) Enzymatic activity of periplasmic DypB_2985_ in the mutants. Absorbance at 420 nm in periplasmic extracts from the parental strain AR_pUPmin_ was used as a blank control to correct for background activity. Activity is expressed as units per liter of culture broth (U/L). (c-d) Periplasmic levels of DypB_2985_ with the N-terminal (c) and C-terminal (d) truncations. Data (b, c and d) are presented as mean values ± standard deviation. n = 3 biological replicates. “**”: *p* < 0.01, “***”: *p* < 0.001 by two-sided Student’s *t*-tes*t*. (e-g) The interaction between Lpp_1528_ and the DypB_2985_ variants. Input cellular lysates and proteins co-precipitated with DypB_2985_ variants were detected by Western blot using anti-Flag (for DypB_2985_ variants) and anti-Myc (for Lpp_1528_) antibodies, respectively. IgG was used as a negative control to assess non-specific binding. Data are representative of two biological replicates.

The deletion of *lpp*_*1528*_ impaired the periplasmic translocation of DypB_2985_ (Fig 3a-3b). Complementation of *lpp*_*1528*_ in the A∆*lpp*_*1528*_ strain restored the periplasmic level of DypB_2985_ to 90% of that observed in AR_2985_ (Figs 3a and E in S1 Appendix), indicating no statistically significant difference between AR_2985_ and A∆*lpp*_*1528*_*::lpp*_*1528*_ (*p* = 0.056). Correspondingly, the periplasmic enzyme activity was also recovered to 95% of the level in AR_2985_, with no significant difference observed (*p* = 0.362, Fig 3b). These results confirm the role of Lpp_1528_ in the translocation of DypB_2985_ to the periplasm.

To elucidate the molecular mechanism by which Lpp_1528_ recognizes DypB_2985_ for periplasmic transport, the recognition regions of DypB_2985_ were systematically investigated. We sequentially truncated 6, 12, 18, and 24 amino acids (aa) at either the N- or C-terminus of DypB_2985_, respectively (Table A in S1 Appendix). We first characterized the protein levels of the truncated DypB_2985_ variants in total cellular lysates. Both ELISA and western blot analyses showed that the levels of N- or C-terminal truncations (6–24 aa) were comparable to those of the wild-type protein (Fig F in S1 Appendix), suggesting that these truncations do not substantially impact protein expression or stability. Moreover, the periplasmic protein levels remained stable following N-terminal truncations of 6–18 aa (*p* > 0.05), but decreased by 42% with a 24-aa truncation (Fig 3c). In parallel, C-terminal integrity proved critical for periplasmic DypB_2985_ levels: removal of just 6 aa reduced protein level by 74%, with progressive reductions of 80–84% observed upon deletions of the 12–24 C-terminal residues (Fig 3d). A subsequent co-IP assay revealed that Lpp_1528_ cannot bind to DypB_2985**Δ**C24_, but retained the ability to interact with DypB_2985**Δ**N24_ and DypB_2985**Δ**C6_ (Fig 3e-3g). These results suggest that the C-terminal region of DypB_2985_ is essential for its interaction with Lpp_1528_. Further mapping localized the Lpp_1528_ recognition site to the C-terminal hydrophobic region (residues 296–320, Fig G in S1 Appendix). Notably, an in vitro pull-down assay did not detect a direct physical interaction between Lpp_1528_ and DypB_2985_ (Fig H(a) in S1 Appendix). However, when A_2985_ cell lysate was used instead of purified Lpp_1528_, DypB_2985_ was retained, consistent with the co-IP results (Fig H(b) in S1 Appendix). These findings indicate that their association may depend on additional cellular factors or specific in vivo conditions.

Lpp_1528_, a membrane lipoprotein in *P*. *putida* A514, contains a Sec signal peptide and was confirmed to localize in the periplasmic space by western blot analysis (Fig I in S1 Appendix). Co-IP and pull-down assays demonstrated that Lpp_1528_ interacts with SecA_4176_, SecB_5893_, and the SecYEG complex, supporting its Sec-dependent export (Figs H(c) and J(a)-J(d) in S1 Appendix). Furthermore, an in vivo interaction between SecYEG and DypB_2985_ was detected, indicating that DypB_2985_ is likely translocated across the inner membrane via the SecYEG translocon, potentially facilitated by Lpp_1528_ (Fig J(e) in S1 Appendix). Notably, the interaction among Lpp_1528_, SecYEG, and DypB_2985_ was transient rather than stable (Fig J(f) in S1 Appendix), consistent with the dynamic cargo-receptor behavior observed in secretory pathways [37,38]. Together, these findings support a model in which Lpp_1528_ functions as a molecular tether that facilitates the translocation of DypB_2985_ across the inner membrane by bridging it to the SecYEG translocon.

### OmpW_4836_ mediates DypB_2985_ sorting into OMVs for extracellular delivery

**Fig 4:**
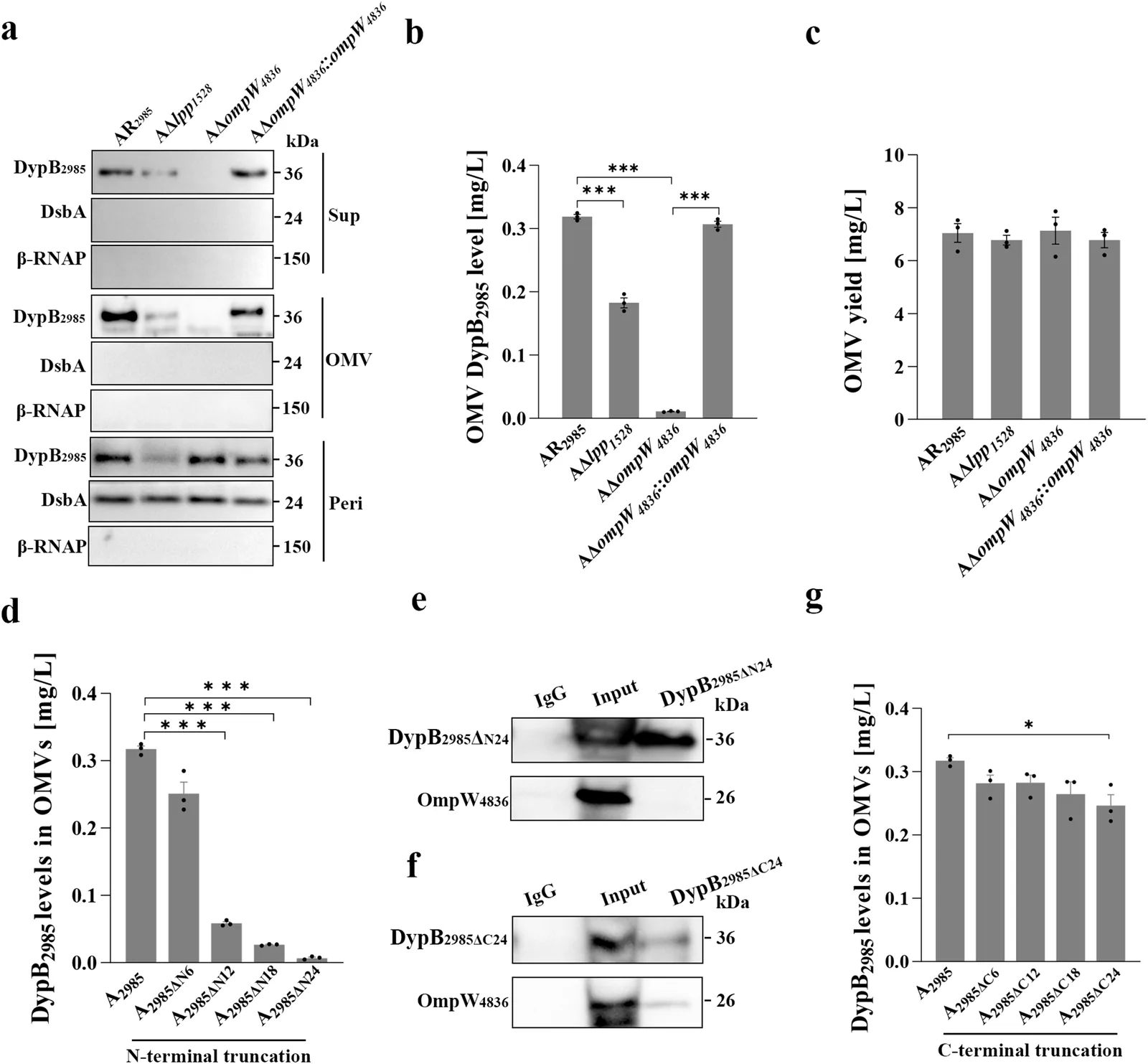
OmpW_4836_ mediates the extracellular delivery of DypB_2985._ **(a)** Western blot analysis of DypB_2985_ protein levels in the periplasm (Peri), OMVs, and culture supernatant (Sup) from the relevant mutants, detected using an anti-His antibody. β-RNAP and DsbA were used as loading controls. AR_2985_ was the reference strain. Data are representative of three biological replicates. **(b)** DypB_2985_ levels in OMVs of the relevant mutants. **(c)** OMV yield in the corresponding mutants, quantified as total protein content per liter of culture broth. **(d, g)** DypB_2985_ levels in OMVs bearing N-terminal (d) and C-terminal (g) truncations. **(e, f)** The interaction between OmpW_4836_ and the N-terminal (e) or C-terminal (f) truncated DypB_2985_ variant. Input lysates and proteins co-precipitated with Flag-tagged DypB_2985_ variants were analyzed by Western blot using anti-Flag (for DypB_2985_) and anti-Myc (for OmpW_4836_) antibodies, respectively. Data (b, c, d, and g) are presented as mean values ± standard deviation. n = 3 biological replicates. “*”: *p* < 0.05, “***”: *p* < 0.001 by two-sided Student’s *t*-tes*t*. Data (e and f) are representative of two biological replicates.

OmpW is not only an OM protein but also a component of OMVs [39]. Deletion of *ompW*_*4836*_ led to a severe reduction in extracellular DypB_2985_ levels in both OMVs and culture supernatant (Fig 4a-4b), despite no significant changes observed in OMV yield and protein composition (Figs 4c and K(a) in S1 Appendix). Complementation of *ompW*_*4836*_ in AΔ*ompW*_*4836*_ restored extracellular DypB_2985_ levels in OMV to those observed in the AR_2985_ strain (Fig 4a-4b). Consequently, DypB_2985_ levels in the culture supernatant were also restored. These results confirm that OmpW_4836_ is essential for OMV-mediated export of DypB_2985_ to the extracellular milieu.

To further identify the region of interaction between DypB_2985_ and OmpW_4836_, we examined the protein levels in OMVs for the 8 recombinant strains with successive truncation at either the N- or C-terminus of DypB_2985_. A_2985**Δ**N12_, A_2985**Δ**N18_, and A_2985**Δ**N24_, with the N-terminal 12–24-aa truncations, significantly reduced the levels of DypB_2985_ in OMVs, by 82–96% (Fig 4d). A_2985**Δ**N24_, in particular, nearly abolished DypB_2985_ expression in OMVs. Consistent with the impaired protein levels in OMVs, deletion of N-terminal residues (24 aa) disrupted the interaction between DypB_2985_ and OmpW_4836_ (Fig 4e). Conversely, A_2985**Δ**C24_, harboring a C-terminal 24-aa truncation, retained the ability to interact with OmpW_4836_ and exhibited a modest 22% reduction in DypB_2985_ level within OMVs (Fig 4f-4g). These results demonstrate that OmpW_4836_ recognizes DypB_2985_ via its N-terminal region (residues 1–24) and mediates its incorporation into OMVs for extracellular delivery.

### Secretion model for *P. putida* DypB_2985_

Deletion of either *lpp*_*1528*_ or *ompW*_*4836*_ did not significantly alter cell growth or compromise cell envelope integrity, as evidenced by similar growth curves and the absence of RNAP (a cytoplasmic marker) and DsbA (a periplasmic marker) in the OMV fractions (Figs 4a and K(b) in S1 Appendix). However, deletion of *lpp*_*1528*_ reduced the periplasmic level of DypB_2985_ by 60%, a reduction comparable to the decrease in extracellular DypB_2985_ levels observed in AΔ*lpp*_*1528*_ relative to the parental strain AR_2985_ (Fig D in S1 Appendix). Conversely, knockout of the *ompW*_*4836*_ gene significantly impaired extracellular DypB_2985_ levels, while periplasmic DypB_2985_ abundance remained largely unchanged (Figs 4a and D in S1 Appendix). These results suggest that Lpp_1528_ facilitates the translocation of DypB_2985_ through the periplasm, whereas OmpW_4836_ is essential for its extracellular delivery.

Notably, DypB_2985_ exhibited enzymatic activity in both cytoplasmic and periplasmic fractions, with activity levels of 22,652 U/L and 2,799 U/L, respectively (Fig L in S1 Appendix). These findings are consistent with previous studies showing that DyP-type peroxidases function both intracellularly and extracellularly, participating in metabolic processes—including intracellular oxidation and hydrolysis, as well as the extracellular degradation of recalcitrant organic compounds [40]. The significant difference in enzymatic activity between the cytoplasmic and periplasmic fractions suggests that only a smaller fraction of DypB_2985_ is translocated across the inner membrane. Given the narrow pore of the SecYEG translocon [41], we hypothesize that DypB_2985_ should be in an unfolded state to pass through the inner membrane. During this process, DnaK prevents the aggregation of the unfolded DypB_2985_. Once in the periplasm, DypB_2985_ either regains or maintains its folded conformation with the assistance of periplasmic chaperones such as SurA [42]. This is supported by our co-IP-MS results, which showed enrichment of both DnaK and SurA (Fig 1c). Furthermore, truncation of the C-terminal 24 aa of DypB_2985_ decreased its periplasmic level from 8.34 mg/L (wild-type) to 1.37 mg/L, representing an 84% reduction (Fig 3d). Surprisingly, the extracellular DypB_2985_ level in OMVs was reduced by only 22% in A_2985**Δ**C24_ (0.25 mg/L vs. 0.32 mg/L, *p* = 0.016, Fig 4g), representing the inherently limited extracellular secretion capacity in *P. putida* A514, a feature previously reported [9,13].

**Fig 5:**
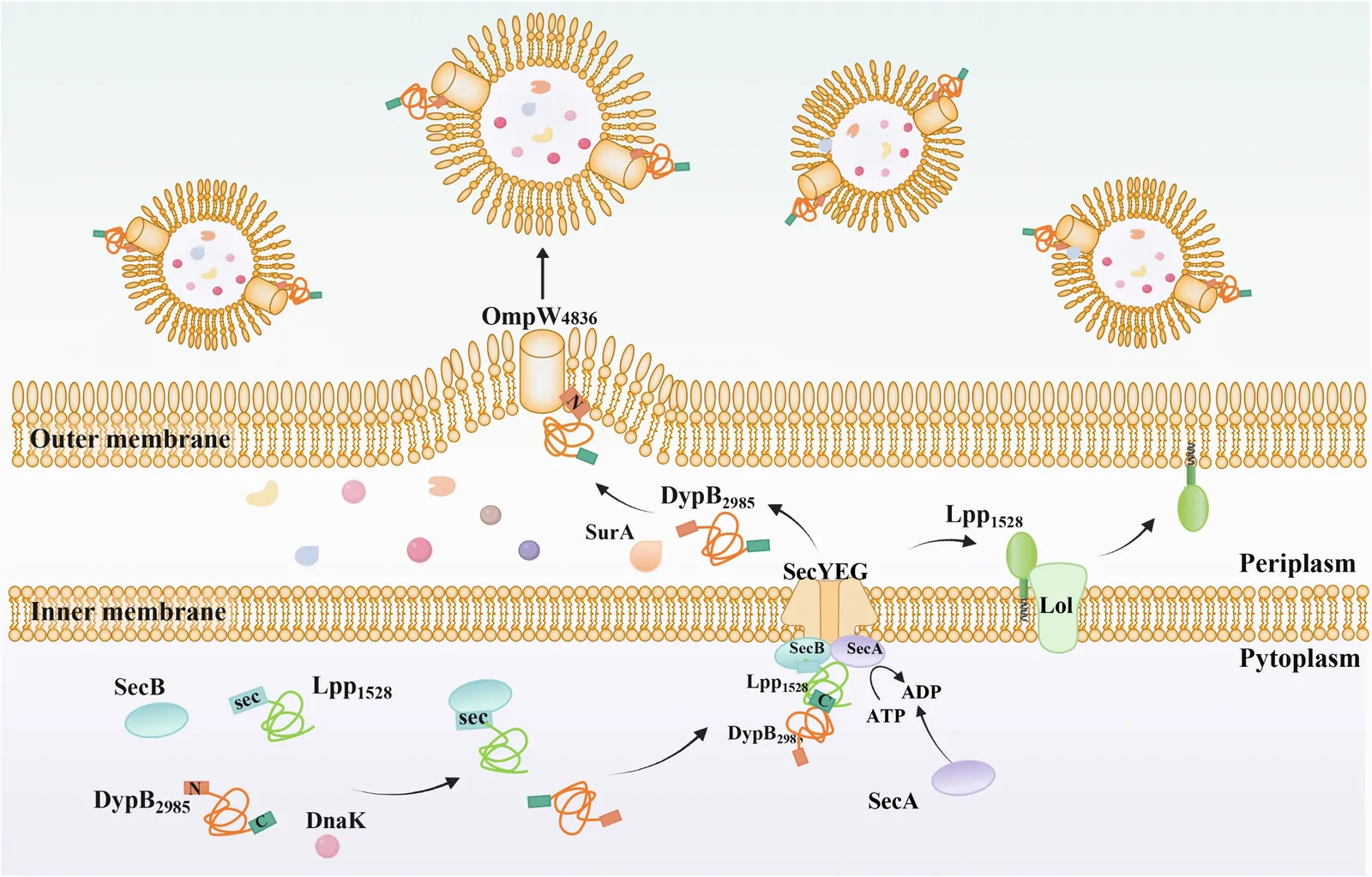
A proposed model for the unconventional secretion of DypB_2985_. Lpp_1528_ interacts with the Sec pathway components, SecB_5893_ and SecA_4176_, to facilitate the translocation of DypB_2985_ across the inner membrane through the SecYEG translocon. Lpp_1528_ recognizes the C-terminal region of DypB_2985_, which remains in an unfolded state and is protected by the chaperone DnaK_4638_, thereby enabling its delivery to the Sec machinery for periplasmic export. Once in the periplasm, DypB_2985_ and Lpp_1528_ are predicted to dissociate. Lpp_1528_ is then targeted to the inner leaflet of the OM via the Lol-dependent lipoprotein trafficking pathway. Meanwhile, DypB_2985_ undergoes folding in the periplasm with assistance from periplasmic chaperones (e.g., SurA), is recognized by OmpW_4836_ via the N-terminal region, and is subsequently sorted to the OMV surface. Finally, DypB_2985_ is released into the extracellular environment upon OMV secretion.

We propose a two-step translocation model for DypB_2985_ secretion in *P. putida* that enables its export in the absence of a canonical signal peptide (Fig 5). First, the chaperone SecB_5893_ recognizes the Sec signal peptide of Lpp_1528_ and delivers it to SecA_4176_. Upon ATP hydrolysis, SecA_4176_ undergoes a conformational change to activate the SecYEG translocon (SecY_4873_, SecE_4907_, and SecG_4625_), allowing Lpp_1528_ to be translocated across the cytoplasmic membrane. Meanwhile, Lpp_1528_ functions as an adapter for DypB_2985_, recognizing the C-terminal region and facilitating its passage through the SecYEG channel into the periplasm. During this translocation, DypB_2985_ is maintained in an unfolded state and protected by the cytosolic chaperone DnaK_4638_. Upon entry into the periplasm, the two transiently interacting proteins, DypB_2985_ and Lpp_1528_, appear to dissociate. Lpp_1528_ is subsequently trafficked to the OM via the Lol-dependent pathway [43]. Concurrently, DypB_2985_ folds into its native conformation with the assistance of periplasmic chaperones, such as SurA. OmpW_4836_ subsequently binds to the N-terminal region of DypB_2985_, mediates its sorting to the surface of OMVs, and thereby enables its release into the extracellular environment along with the vesicles.

## Discussion

Protein secretion, a fundamental process in bacteria, allows proteins to fulfill functions in the extracellular environment, e.g., lignin depolymerization. The classical protein secretion mechanisms in bacteria, where conserved signal peptides play an essential role, have been extensively studied. The N-terminal Sec and Tat signal peptides are recognized by Sec/Tat components for inner membrane translocation [3]. In contrast, the C-terminal secretion signals are commonly distinguished by ABC transporters in type I secretion systems (T1SS) and VirB/D4 in type IV secretion systems (T4SS) for extracellular secretion in a single step, without a periplasmic stopover [44,45]. Consequently, the fate of newly synthesized proteins is believed to be determined by signal peptides. They target the exported components in the cytoplasm and guide them to a specific secretory pathway. Some bacterial proteins lacking any of the known signals/motifs have also been observed in the extracellular milieu, and hence classified as non-classically secreted proteins [46,47]. In contrast to proteins secreted via canonical secretion pathways, the secretion of non-canonical proteins is often mechanism-diverse and context-dependent, with distinct strategies employed across different substrates. To our knowledge, there are several export pathways for non-classically secreted proteins. One is the major autolysin related pathway [48], in which a hydrolase (Atl), possessing peptidoglycan degradation activity, can disrupt the structural integrity of cell wall and thus leads to cell lysis for the subsequent release of intracellular proteins [49]. A second mechanism is the SecA2-dependent system, in which SecA2 recognizes signal peptide-deficient proteins destined for export and mediates their translocation across the inner membrane either via the SecA2-SecY2 pathway or SecA2-only pathway [10]. In a third pathway, the membrane lipoprotein LppX serves as a molecular chaperone to assist in the secretion of xylanases in *Paenibacillus* sp. W-61, which also lacks the classical signal peptide [32]. Interestingly, these pathways are predominantly reported in Gram-positive bacteria. In contrast, far fewer non-classically exported proteins have been identified in Gram-negative bacteria, possibly due to their sophisticated and ubiquitous secretion systems, ranging T0SS to T7SS [50]. Among them, T0SS (i.e., OMVs) packages cytoplasmic/periplasmic proteins without the requirement of signal sequences [51], while T6SS exports a wide range of substrates that display a high diversity in sequence [52]. Our recent study reported that the unconventional DypB_3152_ in *P. putida* is secreted by the crosslinked T6SS and OMV secretion pathways [9]. VgrG and Hcp, the T6SS components, function as effector delivery vehicles, facilitating the translocation of DypB_3152_ through the T6SS baseplate complex. Subsequently, periplasmic DypB_3152_ is incorporated into OMVs and released extracellularly. However, the mechanism of its selective loading into OMVs remains unclear. In this study, we revealed another secretion route for the non-canonical *P. putida* DypB_2985_, involving a coordinated interplay between the Sec and OMV pathways. A functional division of labor underlies this process: Lpp_1528_ facilitates the Sec-dependent translocation of DypB_2985_ into the periplasm, while OmpW_4836_ is specifically responsible for the delivery of OMV-encapsulated DypB_2985_ into the extracellular environment.

Molecular chaperones are important accessory proteins assisting in protein secretion. They help maintain the non-native, translocation-competent conformation of secreted proteins before and during translocation, e.g., DnaJ–DnaK and GroEL–GroES [53,54]. In our study, DnaK_4638_ was enriched in co-IP-MS. Given its well-established role in maintaining protein solubility and proper folding during translocation [27], DnaK_4638_ may help stabilize the unfolded state of DypB_2985_ in the cytoplasm, thereby facilitating its subsequent Sec-dependent translocation. However, DnaK_4638_ does not play a direct role in the translocation of DypB_2985_ itself. Moreover, our study identifies Lpp_1528_ as a key factor in the periplasmic localization of DypB_2985_, functioning as a molecular tether. Lpp_1528_ is a lipoprotein that carries a conserved lipobox (Leu-Ala-Gly-Cys) immediately downstream of the N-terminal Sec signal peptide (Fig I(a) in S1 Appendix). It follows the “+2” rule [55,56] and features a serine residue at position 2, indicating that Lpp_1528_ is likely sorted to the OM. Moreover, Lpp_1528_ was detected in the periplasmic space but not in the extracellular space (Fig I(b) in S1 Appendix). We infer that it is likely anchored to the inner leaflet of the OM via the Lol-dependent pathway (Fig 5), coinciding with the typical localization of lipoproteins in Gram-negative bacteria, which are predominantly found in the inner leaflet of the OM or in the inner membrane [57,58].

Lipoproteins are critical components of the OM with the diverse functions, including protein secretion, folding and localization, sensory processes, adhesion, and maintenance of envelope integrity [59,60]. *Esherichia coli* CsgG, a pore-forming lipoprotein in the type VIII secretion system, forms a secretion channel in the OM to transport amyloids [60]. In addition, LppX in *Paenibacillus* sp. W-61, was previously identified as a molecular chaperone for the proper folding of xylanase. It was hypothesized that LppX induces a conformational change of xylanase to assist the export from the cytoplasmic membrane, although the secretory pathway remains elusive [32]. Here, Lpp_1528_ exhibits 26%-28% sequence identity with LppX/CsgG, aligning with the inherent sequence heterogeneity characteristic of lipoproteins [59]. Our study further extends the molecular tether role of lipoprotein in protein secretion. Lpp_1528_ identifies DypB_2985_ in the cytoplasm through the C-terminal hydrophobic region of DypB_2985_ (Fig G in S1 Appendix). Meanwhile, it can be recognized by the Sec pathway components (e.g., SecB and SecA), through its Sec signal peptide, consistent with the canonical Sec-dependent transport mechanism used by *E. coli* lipoprotein to traverse the inner membrane [61]. Therefore, Lpp_1528_ functions as an adaptor that bridges DypB_2985_ to the Sec translocon for periplasmic transport, despite DypB_2985_ lacking a Sec signal peptide. Such cargo-receptor interactions are typically transient and do not form stable, high-molecular-weight complexes [62]. Notably, in contrast to the direct interaction observed between classically secreted proteins and secretion machinery components [63], such as Lpp_1528_ and SecA_4176_ (Fig H(c) in S1 Appendix), the interaction between Lpp_1528_ and DypB_2985_ is indirect and likely depends on specific cellular contexts or auxiliary factors. Furthermore, deletion of *lpp*_*1528*_ did not completely abolish periplasmic accumulation of DypB_2985_, with approximately 40% of the protein still detected in the periplasm. This observation is consistent with the finding that truncation of the N-terminal 24 aa of DypB_2985_ caused a 42% reduction in its periplasmic levels, despite the truncated variant maintaining interaction with Lpp_1528_. These results suggest that DypB_2985_, which lacks a canonical signal peptide, may employ multiple, redundant secretion pathways to ensure robust periplasmic localization. In addition to the Lpp_1528_-dependent transport that depends on the C-terminal region, an alternative secretion mechanism, potentially involving the N-terminal region (residues 1–24), may also contribute to its efficient targeting to the periplasm under diverse physiological conditions.

The OmpW family is a group of outer membrane porins and is widely distributed in Gram-negative bacteria, where it mediates the transport of small molecules across the OM. These proteins typically fold into an eight-stranded β-barrel to form a narrow hydrophobic pore (~1.3 Å radius) in the membrane for the passage of small hydrophobic molecules (< 660 Da), e.g., polycyclic aromatic hydrocarbons [33]. Notably, a recent study revealed that the OmpW family protein AlkL in *P. putida* GPo1 can reconstruct a barrel extension to generate dynamic lateral exit sites that are large enough to accommodate alkanes (e.g., carvone and octane) with molecular weights of approximately 150 Da [64]. AlkL is located within the operon of alkane utilization, along with monooxygenase (AlkB), reductase (AlkT), dehydrogenases (AlkH and AlkK), and therefore increases the incorporation of alkanes, contributing to improved nutrient acquisition in *P. putida* GPo1 [64,65]. However, its role in mediating the export of proteins larger than 10 kDa via the dynamic lateral exit pathway remains to be elucidated.

OmpW is also a component of OMVs [66]. OMVs encapsulate a diverse and variable cargo, including OM proteins, cytoplasmic and periplasmic proteins, nucleic acids (DNA and RNA), membrane lipoproteins, and phospholipids, whose composition is highly dependent on bacterial species and growth conditions [67]. Current views suggest that the inclusions should be determined by some unknown protein sorting mechanisms, rather than random packaging [68,69]. To overcome the poorly understood sorting rules, protein fusion expression is commonly employed to package a specific protein in OMVs. Our previous study individually fused *Pseudomonas* DypB_3152_ with the OMV components SylB and LolB, and successfully displayed DypB_3152_ on the surface of OMVs [9]. Similarly, green fluorescence protein was enclosed into *E. coli* OMVs, through fused expression with OmpW [66]. Here, our study pinpoints that OmpW specifically sorts DypB_2985_ into *P. putida* OMVs, shedding light on the mechanism of protein sorting in OMVs. It recognizes periplasmic DypB_2985_ through protein-protein interactions involving its N-terminal hydrophobic region (6–24 aa) and facilitates the surface display of DypB_2985_ on OMVs (Fig 5). As previously reported, OMVs act as natural nano-bioreactors that enable efficient lignin bioconversion, with key advantages including high local enzyme concentration, improved substrate accessibility, and enhanced environmental stability [9,70,71]. Our study provides a foundation for engineering OMVs as synthetic nano-bioreactors for biocatalytic applications.

In conclusion, this study reveals a non-classically secretion pathway for the peroxidase DypB_2985_ in *P. putida*. *P. putida* employs the outer membrane proteins to coordinate the classical Sec pathway and OMVs for the export of this unconventional protein. It represents a cost-saving survival strategy that allows bacterial strains to flexibly employ extant systems in response to a dynamic nutrient environment. The lipoprotein connects DypB_2985_ to the canonical Sec pathway, thereby extending the repertoire of accessory proteins. Furthermore, the OMV protein sorting mechanism was explored. OmpW plays a role in sorting DypB_2985_ into OMVs, which provides a promising strategy for engineering OMV cargo composition in the near future.

## Materials and methods

### Bacterial strains and culture conditions

The strains utilized in this study are summarized in Table A in S1 Appendix. *Escherichia coli* DH5α was cultured in Luria-Bertani (LB) medium at 37 °C and 150 rpm, and subsequently used for all molecular cloning procedures. *P. putida* A514 and the relevant strains were cultured either in LB medium or 15 mM glucose-M9 medium at 30 °C, 150 rpm, respectively [13]. The optical densities at 600 nm (OD_600_) were measured to monitor cell growth. All growth experiments were performed with three biological replicates. In addition, recombinant *E. coli* BL21 (DE3) strains B_pGEX_ and B_pGEX1528_ were cultured in LB medium supplemented with 100 μg/mL ampicillin at 37 °C and 150 rpm. When the cultures reached an OD_600_ of 0.6–0.8, 1 mM IPTG (final concentration) was added to induce protein expression. The cultures were then incubated overnight at 16 °C to promote optimal expression of GST or GST-Lpp_1528_. Following induction, cells were harvested by centrifugation at 8,000 × g, 4 °C, 10 min and used for GST pull-down assays.

### Plasmid and mutant construction

All plasmids and primers utilized in this study are listed in Tables C and D in S1 Appendix, respectively. Plasmids were constructed using standard molecular cloning methods and were verified by Sanger sequencing (Applied Biosystems, ThermoFisher). The host strain, ARc9n [9], which showed similar DypB_2985_ protein levels with A_2985_ (Fig B in S1 Appendix), was used for gene deletion experiments. Mutants were constructed based on our previously developed CRISPR/Cas9n-based genome editing method [9,36]. To delete *lpp*_*1528*_, the plasmid pUCP1823 was transformed into ARc9n by electroporation and subsequently cultured in 5 mL LB medium supplemented with 4 mM xylose, 30 μg/mL tetracycline, and 20 μg/mL uracil at 30 °C for 18 h. A 50 μL aliquot of the cell culture was transferred to 5 mL of fresh 15 mM glucose-M9 medium with 4 mM vanillic acid, incubated overnight at 30 °C and 200 rpm for plasmid curing, and spread on M9 medium agar plates. To verify the *lpp*_*1528*_ deletion, colonies without green fluorescence signals were selected for PCR detection and Sanger sequencing. Additional gene knockouts (∆*dnaK*_*4638*_, ∆*ompW*_*4836*_, and ∆*duf4938*_*1524*_) were performed using the same procedures (Table A in S1 Appendix). Each mutant was transformed with the plasmid pUHis2985 to carry a copy of *dypB*_*2985*_ with N-terminal His-tag, generating the following strains: A∆*dnaK*_*4638*_, A∆*ompW*_*4836*_, A∆*lpp*_*1528*_, and A∆*duf4938*_*1524*_.

For the complementation assay, each complementary plasmid (pUH2985–1528 and pUH2985–4836) was electroporated into the corresponding mutant strain and subsequently selected on LB agar plates supplemented with 30 μg/mL gentamicin.

For the gene truncation assay, the DNA fragments of the N-/C-terminal (18, 36, 54, and 72 bp) *dypB*_*2985*_ to be removed were PCR amplified with the A514 genome as a template. The N-terminal His-tag was fused to each truncated *dypB*_2985_ DNA fragment by PCR amplification. Next, the respective fragments were inserted into plasmid pUCP18-Gm, through *Pst* I and *Kpn* I, to construct the corresponding plasmids (Table C in S1 Appendix). They were then transformed into A514 by electroporation, generating the relevant recombinants (Table A in S1 Appendix).

For the GST pull-down assay, pGEX4T-1 and pGEX1528 were transformed into *E. coli* BL21(DE3), and selected on LB agar plates containing 100 μg/mL ampicillin.

### Preparation of the extracellular, periplasmic, and total cellular lysate fractions

Each A514 variant was grown at 30 °C with shaking at 150 rpm in LB medium supplemented with 30 µg/mL gentamicin. 100 mL of the cell culture was centrifuged (8,000 × g, 10 min, 4 °C) at OD_600_ ~ 6.5. The supernatants were collected as the extracellular total proteins, while the cell pellets were used for the isolation of periplasmic proteins and total cellular lysates. Periplasmic proteins were extracted using the osmotic shock method [13]. The cell pellets from 100 mL cell culture were resuspended in 10 mL of buffer A (30 mM Tris–HCl (pH 8.0), 20% (m/v) sucrose, and 1 mM EDTA (pH 8.0)), incubated at room temperature for 20 min, and then centrifuged at 8,000 × g for 10 min at 4 °C. The pellets were subsequently resuspended in ice-cold 5 mM MgSO_4_ buffer (pH 6.0) and incubated at 4 °C for 20 min. The supernatants were collected by centrifugation (8,000 × g, 10 min, 4 °C), as the periplasmic extracts. In addition, the cell pellets from a 100 mL aliquot cell culture were resuspended by ice-cold lysis buffer containing 1 × protease inhibitor cocktail and lysed by sonication (150 W, 1 s on/off for 5 min) [9]. The supernatants were obtained by centrifugation (13,000 × g, 5 min, 4 °C), as total cellular lysates. The protein samples were used for the subsequent co-IP assay and the DypB_2985_ expression assay. Protein concentrations of the relevant samples were measured using the BCA Protein Assay Kit (Thermo, USA, catalog #23225). All experiments were carried out in biological triplicate.

### Purification of recombinant proteins

Each His-tagged recombinant protein, e.g., DypB_2985_ and SecA_4176_, was purified using a Ni-NTA column (Takara, China, catalog #635657) according to previously described protocols [13,36]. Briefly, clarified cell lysates were loaded onto a 5 mL Ni-NTA column that had been pre-equilibrated with binding buffer (20 mM imidazole, 500 mM NaCl, 20 mM Tris-HCl, pH 8.0). Proteins bound to the resin were eluted with elution buffer (50 mM sodium phosphate, 300 mM NaCl, 160 mM imidazole, pH 7.4). The purified proteins were analyzed by 12% (w/v) sodium dodecyl sulfate–polyacrylamide gel electrophoresis (SDS–PAGE) and subsequently collected for downstream applications.

### DypB activity assay

Enzymatic activity was measured using a microplate reader (BioTek, Cytation 5, USA) at 25 °C. DypB activity was assessed by monitoring the oxidation of 5 mM ABTS (2,2′-azino-bis (3-ethylbenzothiazoline-6-sulfonic acid), Aladdin, China, catalog #30931-67-0) in 50 mM acetate buffer (pH 4.5) containing 1 mM H_2_O_2_. Absorbance at 420 nm was recorded (*ε*_420_ = 36,000 M^−1^ cm^−1^) [13]. To account for background enzyme activity, the absorbance value of AR_pUPmin_ (ARc9n strain harboring the pUCP_min_ empty vector) was used as the baseline control (Table A in S1 Appendix). One unit of DypB activity was defined as the quantity of enzyme required to oxidize 1 μmol of ABTS per minute [72]. All experiments were performed in biological triplicate.

### Co-immunoprecipitation MS analysis (co-IP-MS)

A_Flag2985_ was cultured in LB medium at 30 °C until the OD_600_ reached at 6.5. Cells were collected and washed twice with 4 °C phosphate-buffered saline (PBS, pH 7.4) by centrifugation (8,000 × g, 10 min, 4 °C). They were then resuspended in lysis buffer (10 mM Tris pH 7.5, 100 mM NaCl, 0.5% (v/v) Triton X-100, and 100 mM PMSF) and sonicated to extract total cell lysates (150 W, 1 s on/off for 5 min). The cell lysates were incubated with 2 μL anti-Flag antibody (ABclonal, China, catalog # AE005; 1:10,000 dilution) at 4 °C for 16 h. Subsequently, 20 μL protein A/G MagBeads (Yeasen, China, catalog #P2108) were introduced, and rotated at 4 °C and 50 rpm for 4 h to form antibody-protein complexes. Complexes were captured by magnetic adsorption [9]. Parallel incubations were performed using 2 μL mouse IgG HRP-conjugated antibody (ABclonal, China, catalog #AS061; 1:10,000) as the negative control to exclude non-specific binding of target proteins to antibodies or magnetic beads [73,74].

The 20 μL of eluted proteins were separated by 12% SDS-PAGE. The gel was sliced and digested. The resulting peptides were extracted and lyophilized, as previously described [75]. The peptides were resuspended in a solution containing 2% (v/v) acetonitrile and 0.1% (v/v) trifluoroacetic acid (TFA). The peptide mixture was desalted using C18 ZipTip (Millipore, USA) and subjected to proteome analysis via a nano system coupled with a high-resolution mass spectrometer system (Thermo Scientific, USA) [76]. The raw data were searched against the proteome sequence databases of *P. putida* A514 Genome Database [76]. The detailed mass spectrometry protocol is described in the Supplementary Methods. All experiments were performed in three biological replicates.

### Co-immunoprecipitation assay

The extracted cell lysates (1 mg) were incubated with the corresponding antibody (2 μL) and protein A/G MagBeads (20 μL) overnight at 4 °C and 50 rpm, followed by five washes with the extraction buffer. Proteins were eluted from the beads by boiling in SDS loading buffer for 10 min, which were then analyzed by Western blot assay. The antibodies used include anti-Flag antibody (1:10,000 dilution) for detection of N-terminally Flag-tagged DypB_2985_, anti-Myc antibody (ABclonal, China, catalog #AE010; 1:10,000 dilution) for the N-terminally Myc-tagged candidate protein, and anti-His antibody (ABclonal, China, catalog #AE003; 1:10,000 dilution) for the N-terminally His-tagged candidate protein. Mouse IgG HRP-conjugated antibody (ABclonal, China, catalog #AS061; 1:10,000 dilution) was used as a negative control in the IgG group to rule out non-specific binding of target proteins to the antibodies or magnetic beads [77].

### Western blot assay

Each 60 µg protein sample was separated by 12% SDS-PAGE and transferred onto a polyvinylidene fluoride (PVDF) membrane (0.45 µm, Millipore, USA, catalog # ISEQ00010). The membrane was blocked with QuickBlock Blocking Buffer (Beyotime, China, catalog #P0260) for 1 h at room temperature (RT) and then incubated with the corresponding primary antibody overnight at 4 °C [78]. Anti-His antibody (1:10,000 dilution) was used to detect DypB_2985_ with a N-terminal His-tag. In addition, anti-DsbA antibody (Sangon, China, catalog #D190650; 1:2,000 dilution) was employed to detect the disulfide bond formation protein A (DsbA), a well-established periplasmic marker. Anti-RNAP antibody (Boster, China, catalog #BM5368; 1:2,000 dilution) was used to detect the RNA polymerase Beta subunit (β-RNAP), serving as an internal control for total cell lysates and as a negative control to exclude cytoplasmic contamination in the periplasmic fraction and subsequent OMVs. Meanwhile, anti-OmpA antibody (CUSABIO, USA, catalog #CSB-PA359226ZA01EOD; 1:2,000 dilution) was applied to detect OmpA, used as a loading control for OMV surface proteins. Next, the membrane was washed 5 times with TBST buffer (50 mM Tris, 150 mM NaCl, 0.05% (v/v) Tween 20, pH 7.4) and incubated with a secondary antibody (anti-mouse IgG HRP-linked (ABclonal, China, catalog #AS061; 1:10,000 dilution) for 1 hour [79]. Signals were detected using the ECL immunoblotting detection reagent (Bio-Rad, USA, catalog #1705061) with a Chemiluminescence imager (Tanon 5200Multi, Beijing) as previously described [80]. Protein quantification was performed using ImageJ software. The relative expression level of DypB_2985_ was calculated as the ratio of DypB_2985_ protein level to that of the internal standard protein [81]. The quantification experiments were performed in biological triplicate.

### GST pull-down assay

The in vitro interaction between DypB_2985_ and Lpp_1528_ was assessed using a GST pull-down assay according to the manufacturer’s protocol (Thermo, USA, catalog #21516). Briefly, either 200 μg of purified DypB_2985_ or 800 μg of A_2985_ cell lysate was used as the input sample (Sample 1), while 200 μg GST or equivalent amount of GST- Lpp_1528_ was used as the bait (Sample 2). Glutathione agarose (25 μL per reaction) were pre-washed and incubated separately with GST or GST-Lpp_1528_ at 4 °C overnight. After five washes to remove unbound bait proteins, the agarose-bound GST or GST-Lpp_1528_ complexes were incubated with the Sample 1 at 4 °C overnight. Following five additional washes with binding buffer, bound proteins were eluted with 150 μL of 1 × SDS loading buffer (95 °C, 5 min). Eluted proteins and input samples were separated by 12% SDS–PAGE and visualized by Western blotting using anti-GST (ABclonal, China, AE077) and anti-His antibodies. In addition, a GST pull-down assay was performed to assess the interaction between SecA_4176_ and Lpp_1528_ using the same procedure.

### Blue Native PAGE analysis

To investigate the *in vivo* protein complex formation among DypB_2985_, Lpp_1528_, and SecYEG, cell lysates from AΔ*lpp*_*1528*_ and AR_2985_ were analyzed under non-denaturing conditions. Briefly, cells were harvested and lysed in ice-cold lysis buffer containing 1 × protease inhibitor cocktail (Beyotime, China, catalog #P1005) to preserve native protein-protein complexes. Following centrifugation (12,000 × g, 20 min, 4 °C), total protein concentrations in the supernatant were quantified. Equal amounts of protein (20 µg) were mixed with Native Gel Sample Loading Buffer (Beyotime, China, catalog #P0016N) and loaded onto a 4%-13% (w/v) gradient Native PAGE gel (Beyotime, China, catalog #P0545S). Electrophoresis was performed under non-denaturing and non-reducing conditions using Native PAGE Running Buffer (Beyotime, China, catalog #P0556) to preserve the integrity of putative DypB_2985_-containing complexes. Subsequently, proteins were transferred to PVDF membranes and probed with an anti-His antibody to detect DypB_2985_, which carries an N-terminal His-tag, as described in the preceding Western blot assay.

### Preparation of total membrane extracts

A_2985-3152_ strain was cultured in LB medium supplemented with 30 µg/mL gentamicin at 30 °C with shaking at 150 rpm until the OD_600_ reached approximately 1.0. A 100 mL aliquot of the cell culture was centrifuged at 8,000 × g for 10 min at 4 °C. The cell pellet was resuspended in ice-cold lysis buffer containing 1 × protease inhibitor cocktail, followed by sonication for cell lysis (150 W, 1 s on/off, total duration 5 min). The cell lysate was centrifuged at 13,000 × g for 20 min at 4 °C, and the supernatant was collected. The supernatant was then ultracentrifuged at 180,000 × g for 2 h at 4 °C using a Beckman Coulter Type 45Ti rotor. The resulting pellet was collected as the total membrane fraction [82].

### OMVs extraction, purification and composition analysis

OMVs were isolated as previously described [83]. Briefly, cell cultures at the stationary phase were centrifuged at 6,000 × g for 20 min at 4 °C. The supernatant was sequentially filtered through 0.45 µm and 0.22 µm vacuum filters to remove residual cells. The filtrate was then ultracentrifuged at 150,000 × g for 1 h at 4 °C using an Optima L-100XP ultracentrifuge (Beckman Coulter, USA). The resulting pellets, containing OMVs, were resuspended in 1 mL of PBS (pH 7.4). OMVs were subsequently purified using a Bacterial MVs Isolation Kit (Rengen Biosciences, China, catalog # BacMV40–10) [9]. Purified OMVs were examined by a scanning electron microscopy (SEM, FEI Quanta 250 FEG, USA) at 0.45 torr and 30 keV beam accelerating voltage, as previously described [9]. The total protein content of OMVs was quantified using a BCA Protein Assay Kit (Thermo, USA, catalog # 23225). Protein components of OMVs were separated by SDS-PAGE and visualized for detection. Briefly, protein samples were mixed with 5 × SDS loading buffer, denatured by boiling at 95 °C for 10 min, and separated on a 12% SDS-PAGE at a constant voltage of 80 V. Following electrophoresis, the gel was stained with 0.1% Coomassie Brilliant Blue G-250 (in 25% isopropanol and 10% acetic acid) for 1 h, and then destained with a solution containing 5% ethanol and 10% acetic acid until distinct protein bands became visible.

### The location of DypB_2985_ in OMVs

Freshly OMVs (20 µg) were extracted from A_2985-3152_ and incubated with 0.1 mg/mL proteinase K at 37 °C for 20 min to digest surface proteins of OMVs. Additionally, 1% Triton 100 was used to disrupt the OMV membrane, and then proteinase K was used to digest the total proteins in the OMVs. The treated samples were analyzed by Western blot assay, using OmpA (a OMV surface protein [20]) and DypB_3152_ (an OMV-resident protein [9]) as the controls. All experiments were performed with two biological replicates.

### ELISA quantification of DypB_2985_ levels

The total cell lysates, periplasmic extracts and OMVs were collected from the indicated A514 strains grown in LB medium, as described above. To measure the DypB_2985_ protein levels in the periplasmic and OMVs samples, ELISA assay was performed by the His tag ELISA Detection Kit (GenScript, China, catalog # L00436), according to the manufacturer’s protocol [84]. All experiments were performed in biological triplicate.

### Statistical analysis

Statistical differences between two groups were evaluated via two-tailed unpaired Student’s *t*-tests. A significance level of *p* < 0.05 was considered statistically significant, *p* < 0.01 was highly significant, and *p* < 0.001 represented highly statistically significant.

## Supporting information

S1 Appendix Fig A Validation of OMV purity in *P. putida* A514. (a) Scanning electron microscopy (SEM) image of isolated OMVs. (b) Western blot analysis of subcellular fractions. β-RNAP was used as a loading control for total cellular lysate; DypB_3152_ served as a control for OMV-resident proteins; and SecY was used as a control for membrane-associated proteins. Their expected localization patterns validate the fractionation integrity. TM: total membrane extracts. Sup: supernatant. Data are representative of two independent biological replicates. Fig B Western blot analysis of DypB_2985_ across subcellular fractions in A_2985_ and AR_2985_: whole-cell lysate (Cell), periplasm (Peri), supernatant (Sup), and OMVs. β-RNAP and DsbA were used as the protein expression controls. Data are representative of two independent biological replicates. Fig C Relative DypB_2985_ levels (a) and enzymatic activity (b) in the periplasm of the relevant mutants. Relative DypB_2985_ level: the periplasmic DypB_2985_ protein abundance normalized to periplasmic DsbA level. Protein levels were analyzed by Western blot densitometry using ImageJ software. For enzymatic activity measurements, the absorption at 420 nm of periplasmic extracts from strain AR_pUPmin_ was used as a blank control to subtract background activity. DypB_2985_ activity is expressed as units per liter (U/L), where one unit corresponds to the activity in 1 L of culture broth. Data are presented as mean values ± standard deviation of three biological replicates. “***”: *p* value < 0.001 by two-sided Student’s *t*-test. Fig D Comparisons of DypB_2985_ protein levels between the mutants and AR_2985_. DypB_2985_ levels were quantified by Western blot densitometry using ImageJ software. Ratio: DypB_2985_ level in each mutant relative to that in AR_2985_. Data are presented as mean values ± standard deviation of three biological replicates. Cell: the total cellular lysate, Peri: periplasmic fraction, Sup: supernatant fraction. “***”: *p* value < 0.001 by two-sided Student’s *t*-test. Fig E Relative periplasmic DypB_2985_ levels in Δ*lpp*_*1528*_ mutants. DypB_2985_ levels were normalized to periplasmic DsbA as a loading control. Protein levels were quantified by Western blot densitometry using ImageJ software. Data are presented as mean values ± standard deviation of three biological replicates. “***”: *p* value < 0.001 by two-sided Student’s *t*-test. Fig F Functional impact of DypB_2985_ truncations on protein expression and stability. (a-b) ELISA-quantified expression levels of N-terminal (a) and C-terminal (b) truncated DypB_2985_ variants in total lysates. Data are presented as mean values ± standard deviation. n = 6 biological replicates. (c-d) Western blot analysis of protein stability for N-terminal (c) and C-terminal (d) truncations. β-RNAP was used as the loading control. Representative images from two independent biological replicates are shown. Fig G DypB_2985_ sequence analysis. Scatter plot of hydrophobic scores for DypB_2985_ using Expasy-ProtScale. The y-axis represents hydrophobicity score with positive values indicating hydrophobicity and negative values indicating hydrophilicity. The truncated DypB_2985_ amino acid sequences are shown with dashes denoting deleted residues and asterisks (*) denoting stop codons. Fig H Interaction of Lpp_1528_ with DypB_2985_ or SecA_4176_ assessed by GST pull-down assay. Purified His-DypB_2985_ (a), cell lysate from A_2985_ strain (b), and purified His-SecA_4176_ (c) were individually incubated with GST or GST-Lpp_1528_. The initial samples (input) and bound proteins (pull-down fractions) were analyzed by Western blotting using anti-GST or anti-His antibodies, respectively. All blots were independently repeated twice with consistent results. Fig I Lpp_1528_ domain structure and localization analysis. (a) An N-terminal Sec signal peptide (in green) targets Lpp_1528_ to export via the Sec pathway. Lipoprotein signal peptidase (Lsp) recognizes the lipobox (Leu-Ala-Gly-Cys) residues and cleaves the signal peptide. The N-terminal region of the mature lipoprotein contains an outer membrane sorting signal (in blue) at position 2. The C-terminal portion of the polypeptide (in orange) assumes a fold specific function (e.g., hydrolase). (b) Western blot analysis of the Lpp_1528_ protein levels in the whole cellular lysate (Cell), periplasm (Peri), supernatant (Sup), and OMVs in A_2985-1528_, where A_2985_ carries the *lpp*_*1528*_ gene with a N-terminal Myc-tag on the pUCP18 plasmid. Lpp_2985_ was detected with anti-Myc antibody. β-RNAP and DsbA were used as the protein expression controls. Data are representative of two independent biological replicates. Fig J DypB_2985_ is exported through the SecYEG translocon. (a) The gene cluster for Sec components in *P. putida* A514. (b-d) The interaction of SecA_4176_ (b), SecB_5893_ (c), and SecYEG (d) with Lpp_1528_. (e) The interaction between SecYEG and DypB_2985_. The initial cellular lysates (Input) and reserved proteins were detected by Western blot with anti-His and anti-Myc antibodies, respectively. IgG represents the negative control. Data (b-e) are representative of two biological replicates. (f) Investigation of the protein complex formation among DypB_2985_, Lpp_1528_, and SecYEG in AR_2985_. The putative DypB_2985_-containing complexes were separated by Blue Native PAGE and detected by Western blotting using an anti-His antibody specific to the N-terminal His-tag of DypB_2985_. A∆*lpp*_*1528*_ was used as a negative control. Fig K OMV protein profiles and growth curves of indicated mutant strains. (a) SDS-PAGE analysis of OMV protein composition from the indicated mutants. Proteins were separated by SDS-PAGE and stained with Coomassie Brilliant Blue. The gel displays the relative abundance and distribution of OMV-associated proteins across strains. Molecular weight markers (M, in kDa) are shown on the left. (b) Cell growth curves of A_2985_, A∆*lpp*_*1528*_, and A∆*ompW*_*4836*_ in LB medium. Fig L Intracellular and periplasmic DypB_2985_ activity in AR_2985_. DypB_2985_ activity was measured in both the intracellular (Intra) and periplasmic (Peri) fractions of AR_2985_. Strain AR_pUPmin_ was used as a blank control to subtract background activity. Data are presented as mean values ± standard deviation of three biological replicates. “***”: *p* value < 0.001 by two-sided Student’s *t*-test. Table A Strains used in this study. Table B Flag-DypB_2985_ interacted proteins by coIP-MS assay.
(DOCX)

S1 Table DypB2985 interacted proteins by coIP-MS assay. Table C Plasmids used in this study. Table D Primers used in this study.
(XLSX)

S1 Raw Gel S1 Raw Gel 1 Uncropped Western blots corresponding to Figs 1 and 2a-2d. Associated figures are shown above the blots and the cropped regions are shown by red boxes. All blot images in this study were acquired using a Tanon 5200 chemiluminescent imaging system. S1 Raw Gel 2 Uncropped Western blots of Fig 2e. Associated figures are shown above the blots and the cropped regions are shown by red boxes. All blot images in this study were acquired using a Tanon 5200 chemiluminescent imaging system. S1 Raw Gel 3 Uncropped Western blots of Fig 3. Associated figures are shown above the blots and the cropped regions are shown by red boxes. All blot images in this study were acquired using a Tanon 5200 chemiluminescent imaging system. S1 Raw Gel 4 Uncropped Western blots of Fig 4. Associated figures are shown above the blots and the cropped regions are shown by red boxes. All blot images in this study were acquired using a Tanon 5200 chemiluminescent imaging system. S1 Raw gel 5 Uncropped Western blots of Figs A, B, and F in S1 Appendix. Associated figures are shown above the blots and the cropped regions are shown by red boxes. All blot images in this study were acquired using a Tanon 5200 chemiluminescent imaging system. S1 Raw gel 6 Uncropped Western blots of Fig H in S1 Appendix. Associated figures are shown above the blots and the cropped regions are shown by red boxes. All blot images in this study were acquired using a Tanon 5200 chemiluminescent imaging system. S1 Raw gel 7 Uncropped Western blots of Figs I and J in S1 Appendix. Associated figures are shown above the blots and the cropped regions are shown by red boxes. All blot images in this study were acquired using a Tanon 5200 chemiluminescent imaging system.
(PDF)

S1 Data Source data are provided in S1 Data.xlsx. (XLSX)
