# Supplementary material for: Outer membrane proteins mediate unconventional secretion of Pseudomonas peroxidase through crosstalk between the Sec pathway and outer membrane vesicles
Source: PLoS Genet. 2026 Sep 15;22(9):e1012311. doi: 10.1371/journal.pgen.1012311 (PMC13592688; doi:10.1371/journal.pgen.1012311)

S1 Raw Gel

S1 Raw Gel 1 Uncropped Western blots corresponding to Fig 1 and Fig 2a-2d. Associated figures are shown above the blots and the cropped regions are shown by red boxes. All blot images in this study were acquired using a Tanon 5200 chemiluminescent imaging system.

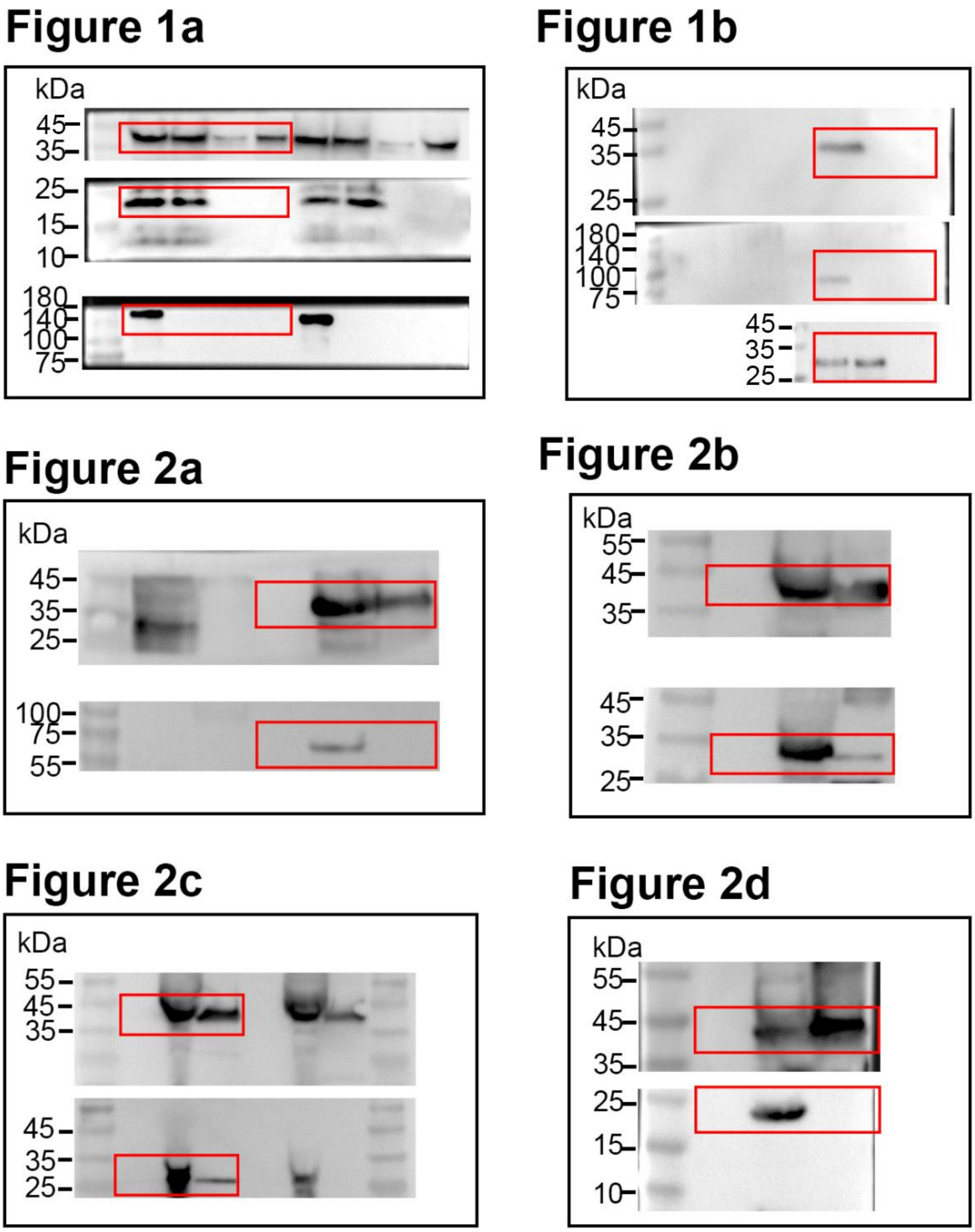

S1 Raw Gel 2 Uncropped Western blots of Fig 2e. Associated figures are shown above the blots and the cropped regions are shown by red boxes. All blot images in this study were acquired using a Tanon 5200 chemiluminescent imaging system.

Figure 2e

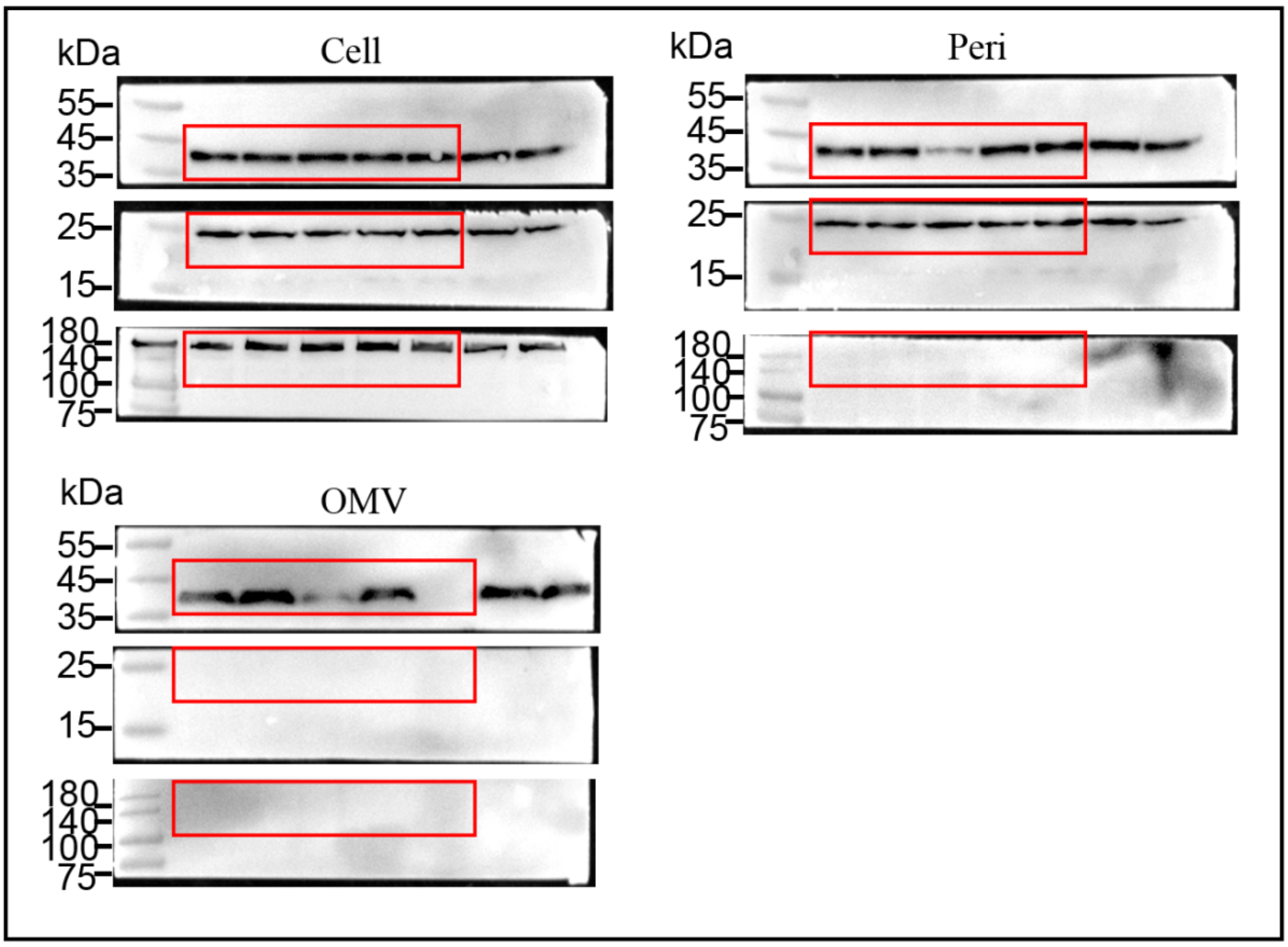

S1 Raw Gel 3 Uncropped Western blots of Fig 3. Associated figures are shown above the blots and the cropped regions are shown by red boxes. All blot images in this study were acquired using a Tanon 5200 chemiluminescent imaging system.

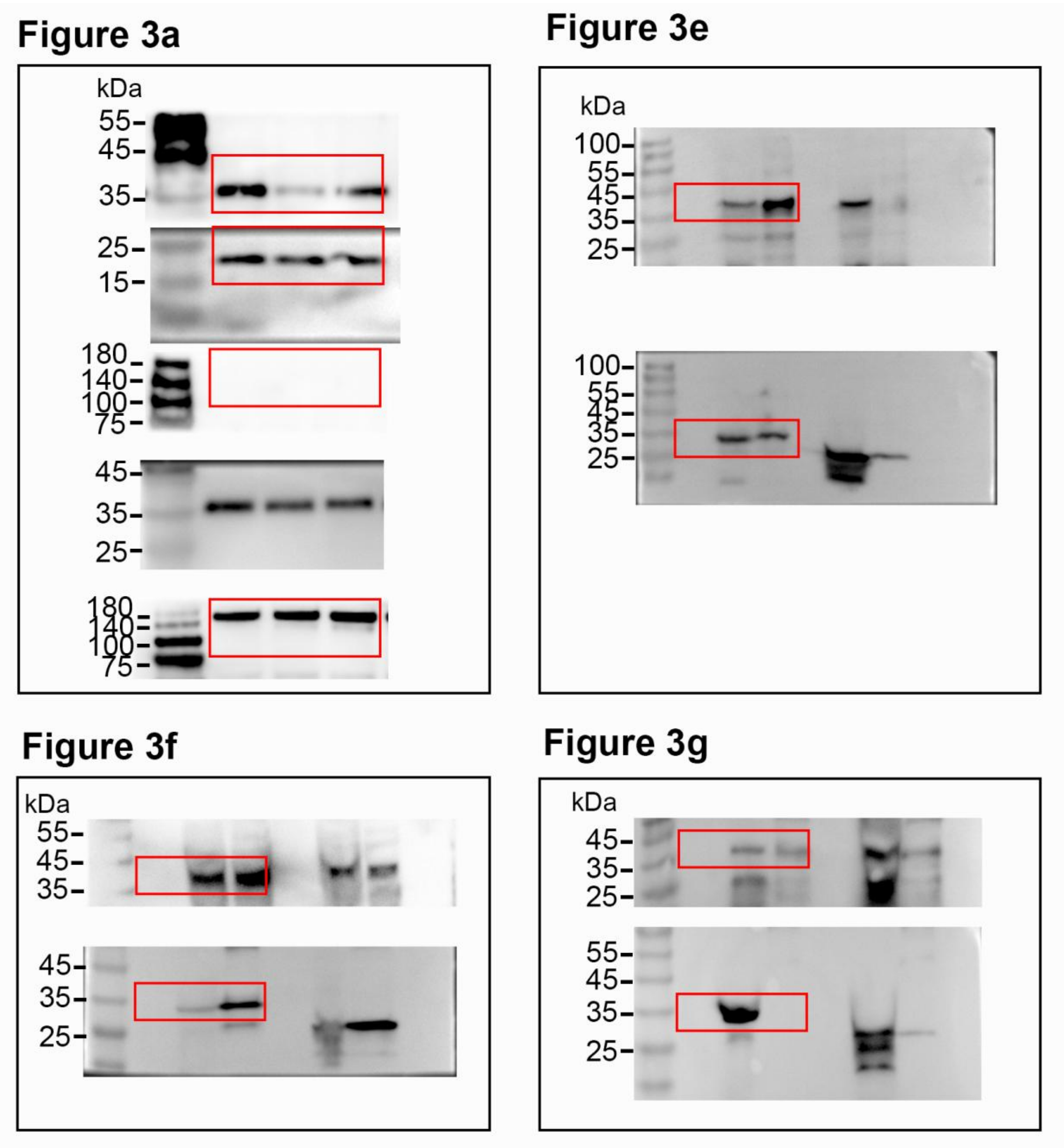

S1 Raw Gel 4 Uncropped Western blots of Fig 4. Associated figures are shown above the blots and the cropped regions are shown by red boxes. All blot images in this study were acquired using a Tanon 5200 chemiluminescent imaging system.

Figure 4a

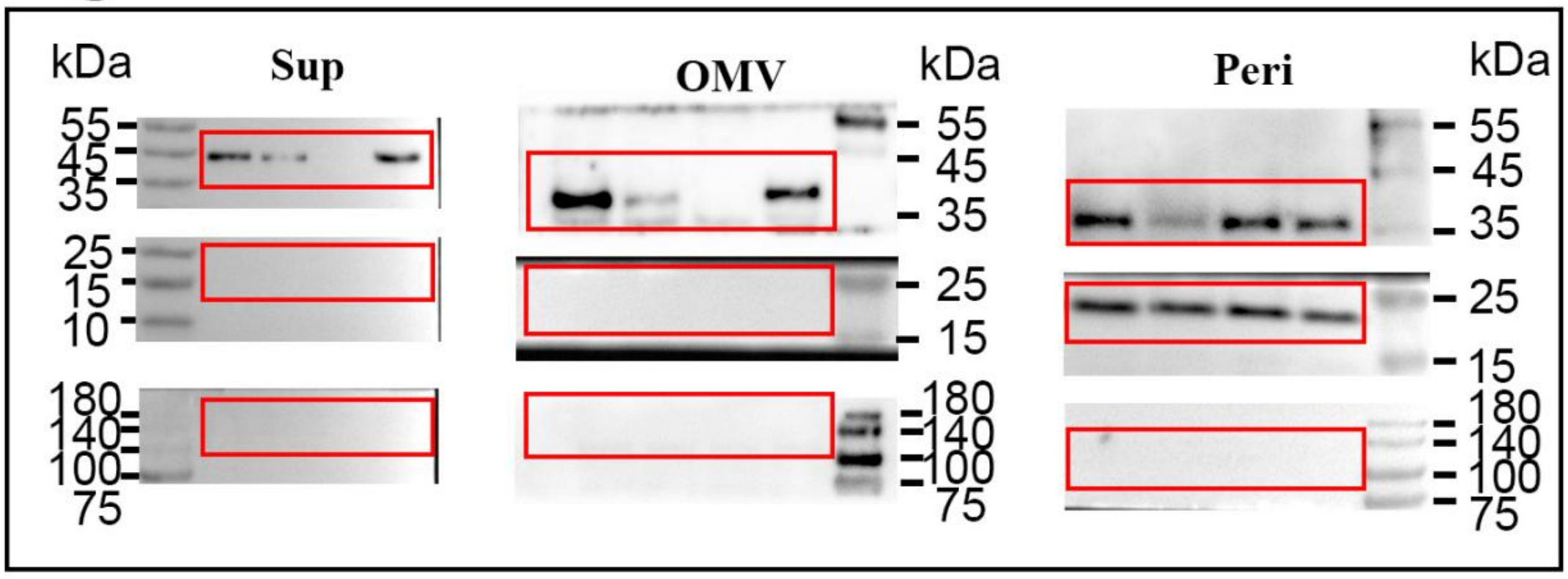

Figure 4e

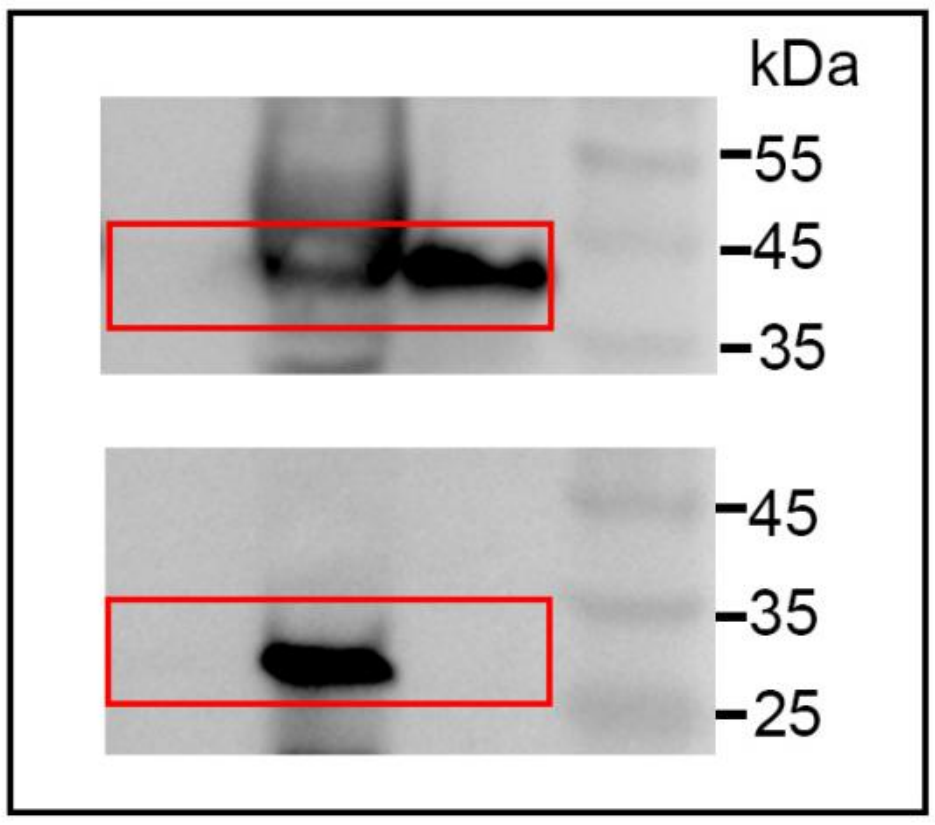

Figure 4f

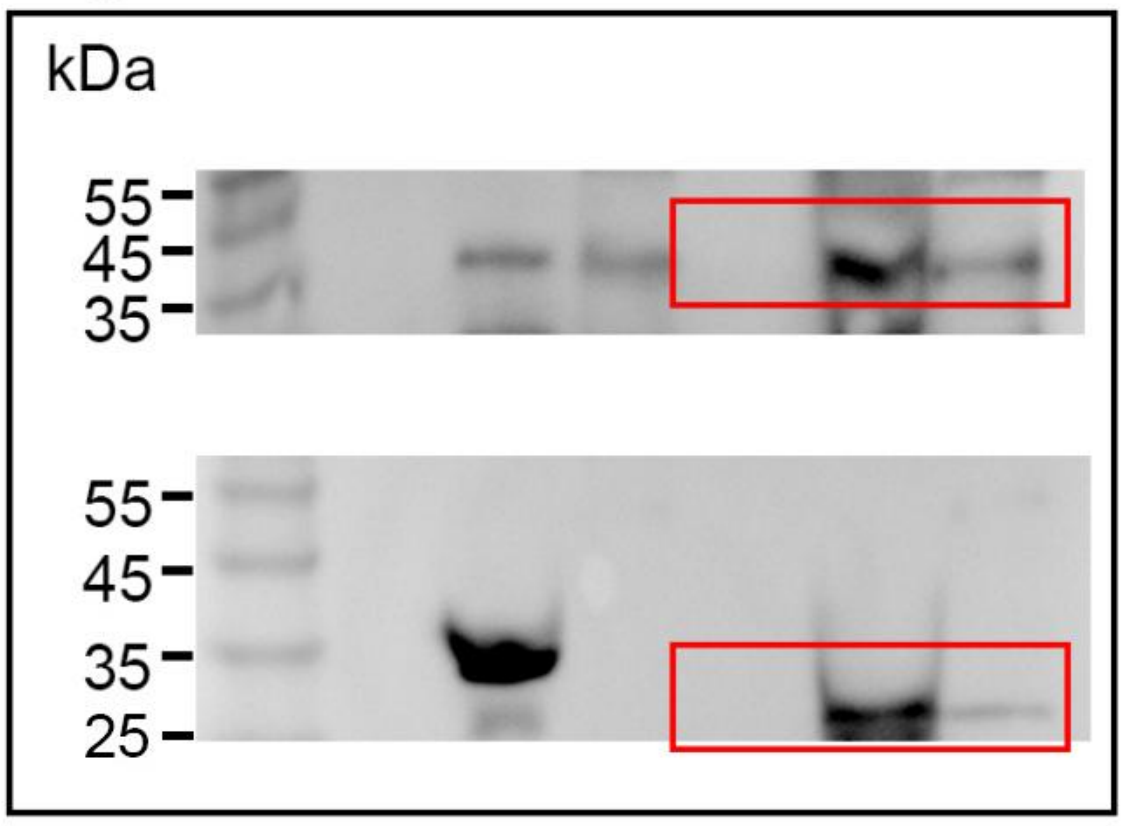

S1 Raw Gel 5 Uncropped Western blots of Figs A, B, and F in S1 Appendix. Associated figures are shown above the blots and the cropped regions are shown by red boxes. All blot images in this study were acquired using a Tanon 5200 chemiluminescent imaging system.

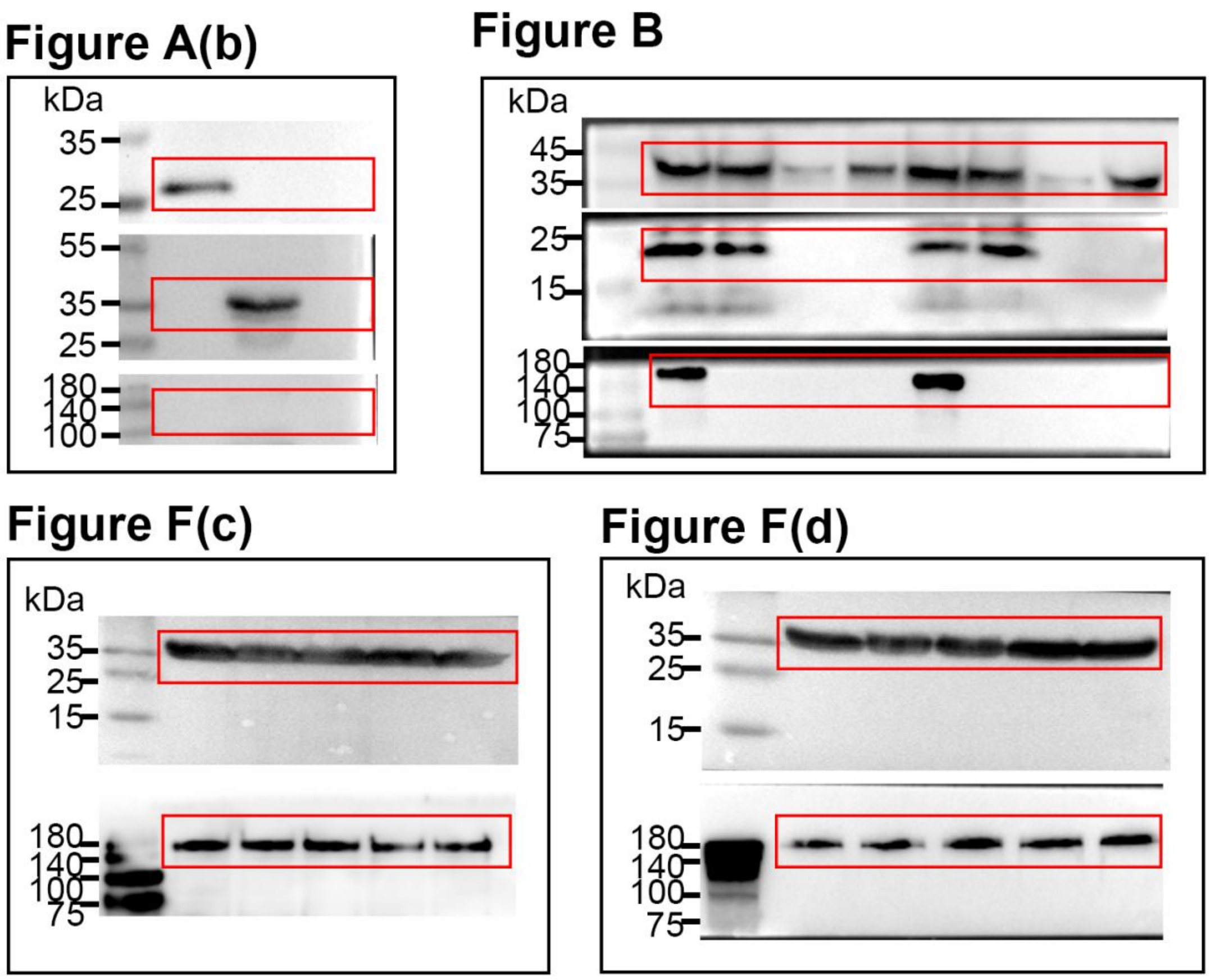

S1 Raw Gel 6 Uncropped Western blots of Fig H in S1 Appendix. Associated figures are shown above the blots and the cropped regions are shown by red boxes. All blot images in this study were acquired using a Tanon 5200 chemiluminescent imaging system.

Figure H(a)

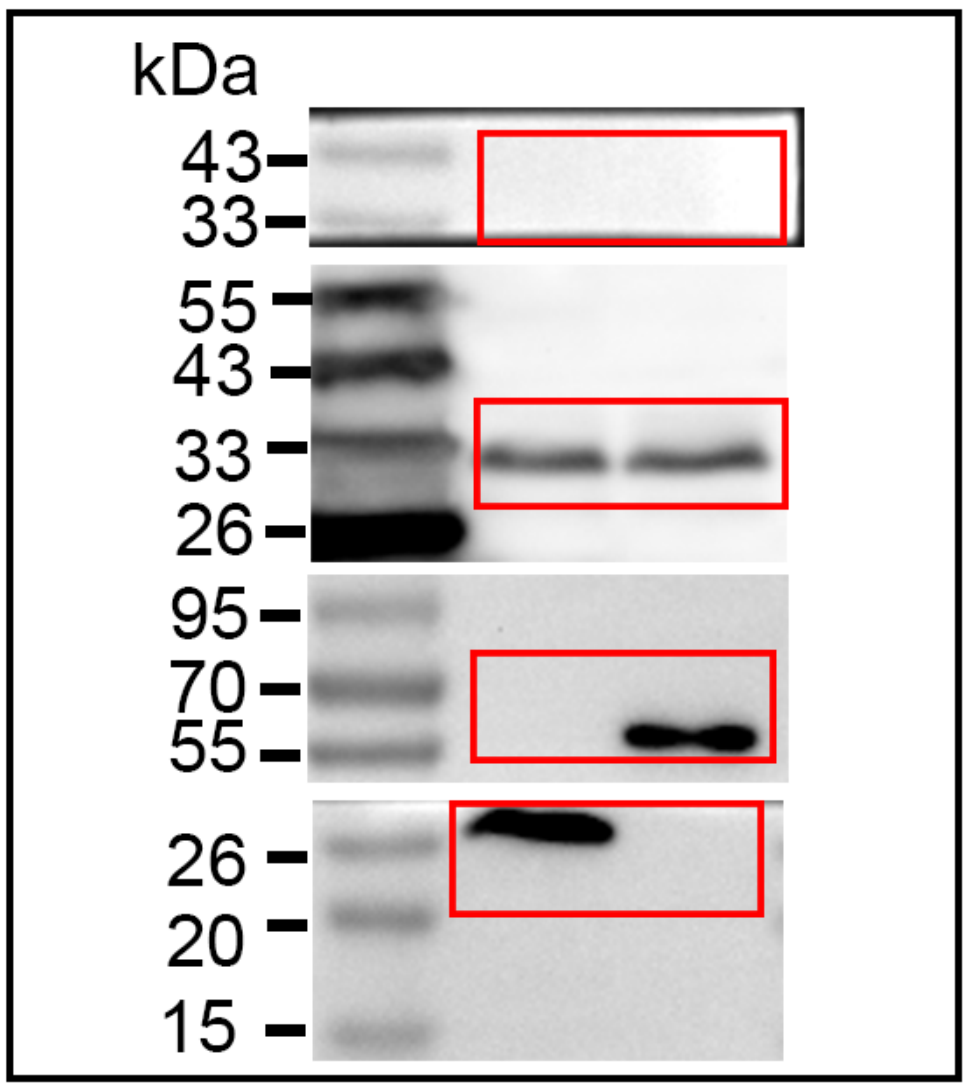

Figure H(b)

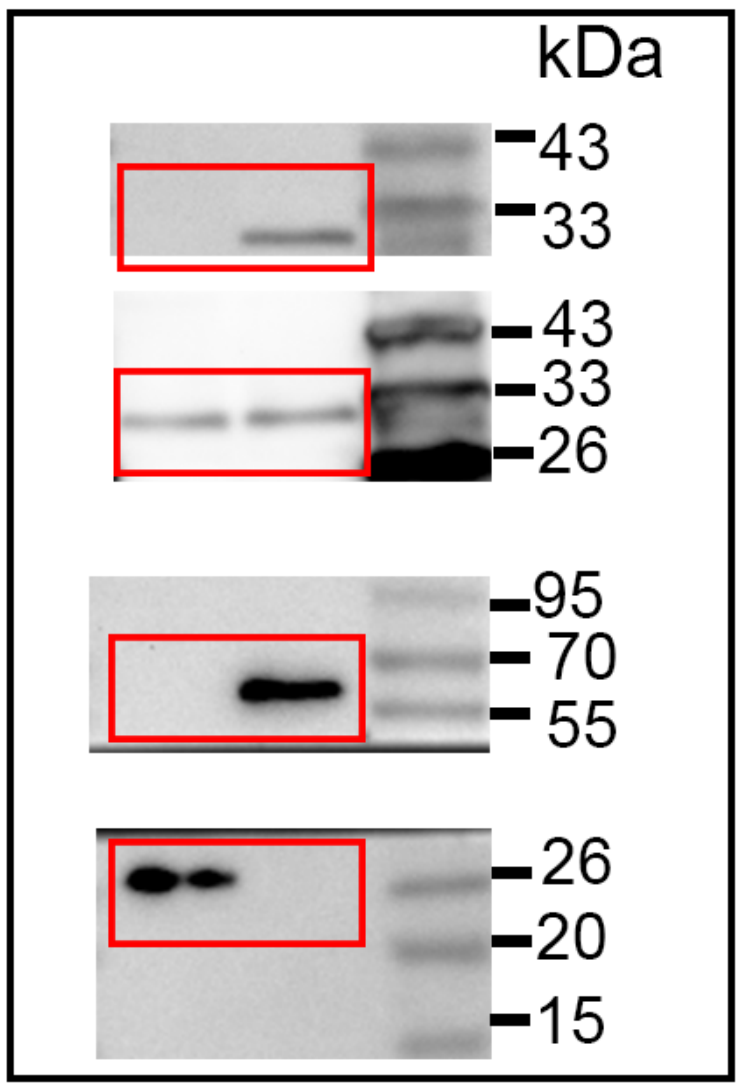

Figure H(c)

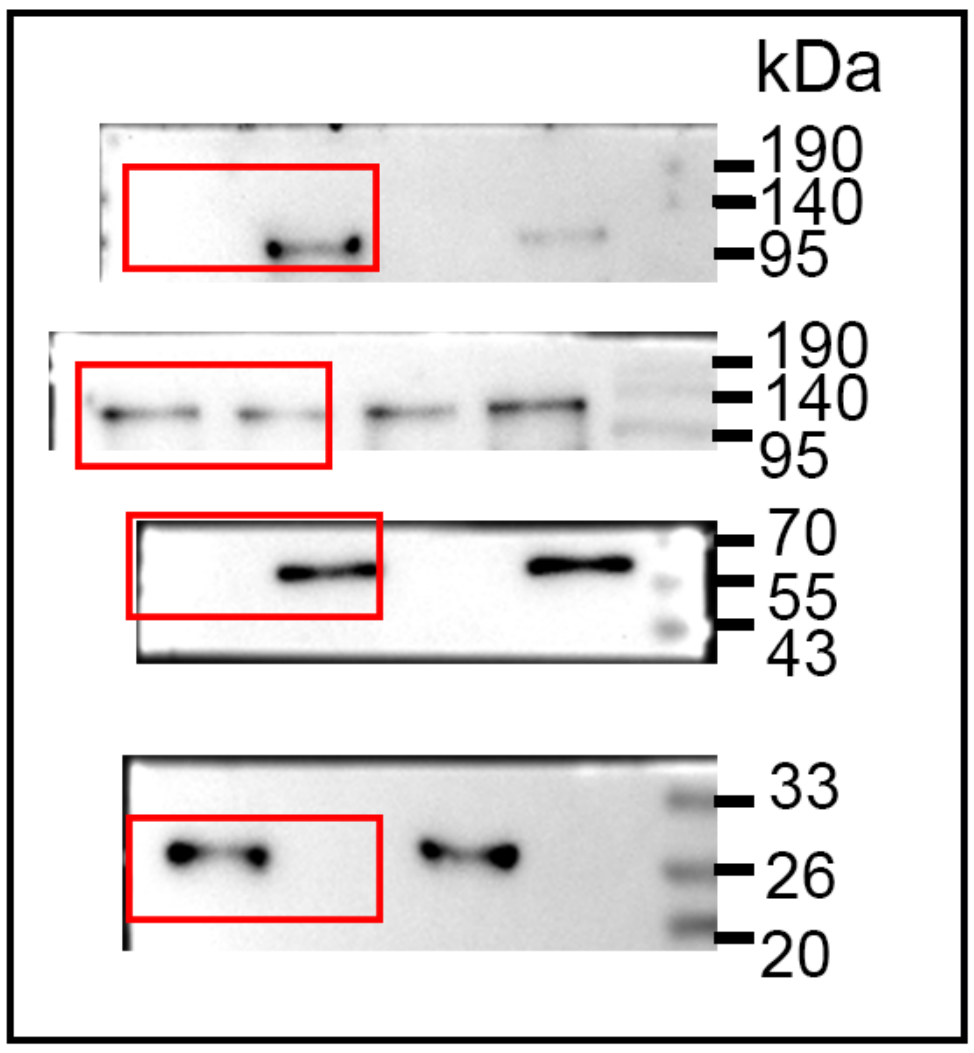

S1 Raw Gel 7 Uncropped Western blots of Figs I and J in S1 Appendix. Associated figures are shown above the blots and the cropped regions are shown by red boxes. All blot images in this study were acquired using a Tanon 5200 chemiluminescent imaging system.

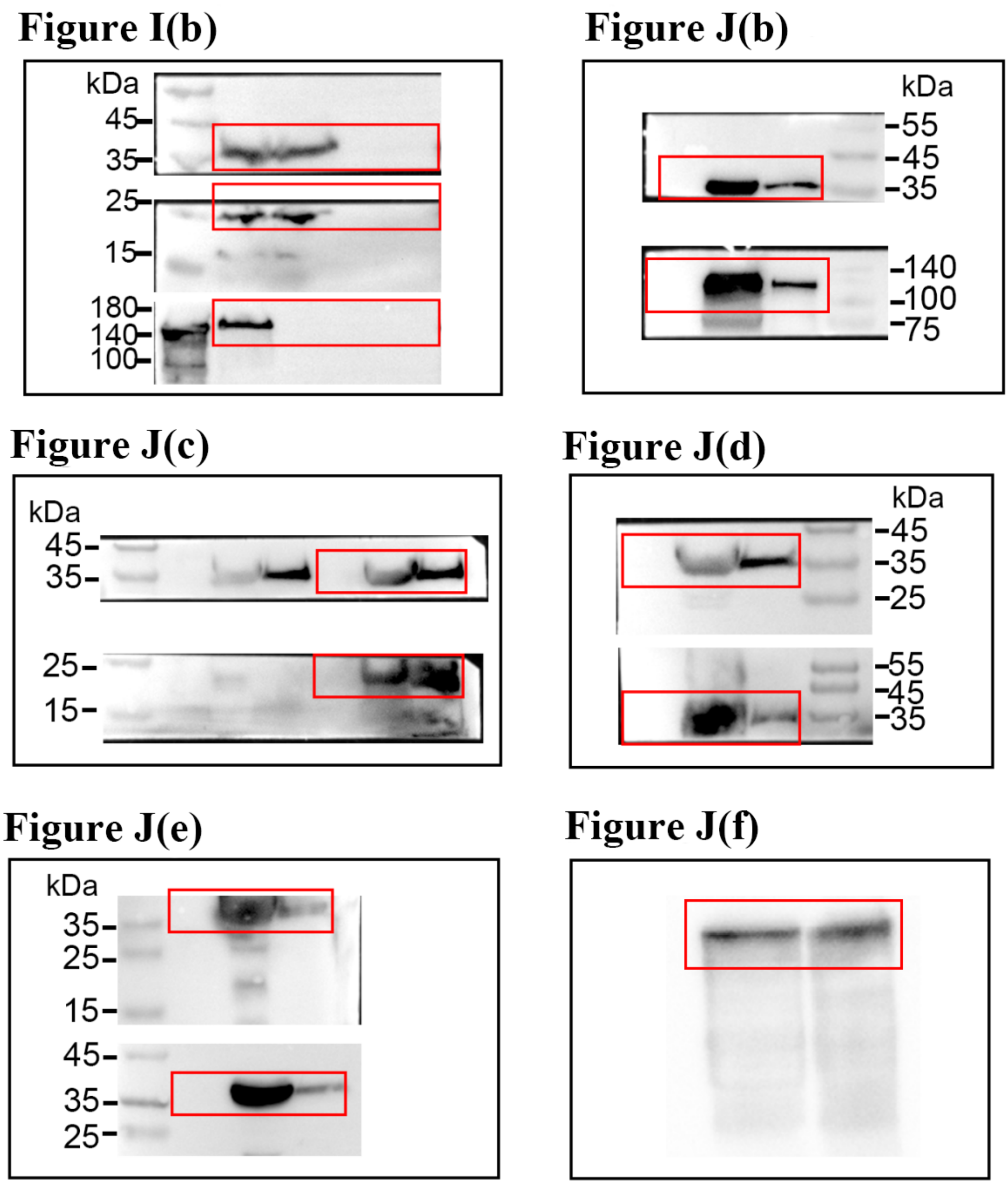

Supplement: Supplementary file 1 [file pgen.1012311.s003.pdf]
