# Supplementary material for: Outer membrane proteins mediate unconventional secretion of Pseudomonas peroxidase through crosstalk between the Sec pathway and outer membrane vesicles
Source: PLoS Genet. 2026 Sep 15;22(9):e1012311. doi: 10.1371/journal.pgen.1012311 (PMC13592688; doi:10.1371/journal.pgen.1012311)
Supplement: Supplementary file 2 [file pgen.1012311.s001.docx]

**Supplementary Information for**

Outer membrane proteins mediate unconventional secretion of *Pseudomonas* peroxidase through crosstalk between the Sec pathway and outer membrane vesicles

Congying Liang^1&^, Wenping Zhu^1&^, Lu Lin^1, 2, 3*^

1. Institute of Marine Science and Technology, Shandong University, Qingdao, China

2. Southern Marine Science and Engineering Guangdong Laboratory (Zhuhai), Zhuhai, China

3. Shandong Key Laboratory of Intelligent Marine Engineering Geology, Environment and Equipment, Qingdao, China

^&^ These authors contributed equally to this work

* linlu2019@sdu.edu.cn (L.L.)

**This file includes:**

Supplementary methods

Supplementary Figures A to L

Supplementary Tables A to D

References

**Other supplementary materials for this manuscript include the following:**

S2_Rawgel

**Supplementary** **methods**

**Plasmid construction**

Plasmid pUCP18-24 was constructed to delete the *lpp_1528_* gene. The DNA fragment of P_trc_::*lpp_1528_* sgRNA was amplified by PCR. The 0.5 kb upstream and downstream homologous arms of *lpp_1528_* were amplified from the A514 genome using the primers 1528 UD-F (*Xba I*)/1528 UD-R (P*pyrF*) and 1528 DD-F (*pyrF*)/1528 DD-R (*Sac* I), respectively (Table D in S1 Appendix). Subsequently, they were spliced to produce a 1 kb homologous segment by overlap extension PCR. The DNA fragment (P_trc_::*lpp_1528_* gRNA) was digested by *Pst* I and *EcoR* I, while the 1 kb homologous arm fragment was digested by *Xba* I and *Sac* I. Meanwhile, the DNA fragment of P_van_::gRNA was digested from pUCP18-2 (1) by *EcoR* I and *Xba* I. The three fragments were inserted into the pUCP18 vector through *Pst* I and *Sac* I, generating the plasmid pUCP18-24. Similarly, pUCP18-23, pUCP18-25, and pUCP18-26, which were used to delete *duf4938_1524_*, *dnaK_4638_* and *ompW_4836_*, respectively, were constructed using the same procedure (Table C in S1 Appendix)*.* All plasmids were verified by DNA sequencing for subsequent experiments.

pUFlag2985 was constructed to screen for proteins that interacted with DypB_2985_ in coIP-MS assay. The open reading frame (ORF) fragment for the *dypB* gene (PputA514_2985) was amplified by PCR with the primers 2985 (*Afl* II)-F and 2985 (*Kpn* I)-R (Table D in S1 Appendix). Through overlap extension PCR, it was fused with Flag to generate the Flag2985 DNA fragment, and was then ligated into the plasmid pUHis2985 through *Xba* I and *Kpn* I, constructing the vector pUFlag2985. In addition, DNA fragments of *lpp_1528_* with an N-terminal Myc-tag and Ph3 promoter were fused together by overlap extension PCR. The resulting fragment was then digested and ligated into the plasmid pUFlag2985 through *Bln* I and *Xho* I, generating the vector pU2985-1528. Similarly, pU2985-1524, pU2985-4638, pU2985-4836, pU4176-1528, pU5893-1528, pUSecYEG-2985, and pUSecYEG-1528 were constructed using the same procedure (Table C in S1 Appendix).

pUH2985-1528 was constructed for the *lpp_1528_* complementation assay. The open reading frame (ORF) fragments for the *dypB* gene (PputA514_2985) and *lpp* (PputA514_1528) gene were amplified by PCR using the following primer pairs: his-2985 F (*Xba* I) and his-2985 R (*Pst* I) for *dypB* and 1528 (*Xho* I)-F and 1528 (*Bln* I)-R for *lpp* (Table D in S1 Appendix). Through overlap extension PCR, they were fused with P_min_ and Ph5 promoters to generate P_min_-2985 and Ph5-1528 DNA fragments, respectively. The DNA fragments were then digested with *Hind* Ⅲ/*Xba* I and *Xba* I/*Bam*H I, respectively, and then were ligated into pUCP18 (Table C in S1 Appendix). Similarly, pUH2985-4836 was constructed using the same procedure.

For the truncated DypB_2985_ assay, the DNA fragments of the removal N-/C-terminal (18-72 bp) *dypB*_2985_ with a N-terminal His-tag were PCR amplified with the A514 genome as a template, and were inserted into the plasmid pU2985-1528 and pU2985-4836 through *Spe* I and *BamH* I to construct the corresponding plasmids pU2985(C6)-1528, pU2985(C24)-1528, pU2985(N24)-1528, pU2985(C6)-4836, pU2985(C24)-4836, and pU2985(N24)-4836 (Table C in S1 Appendix).

pGEX1528 was constructed for the GST pull-down assay. The open reading frame (ORF) fragment of the *lpp* gene (PputA514_1528) was amplified by PCR using the primers 1528-F (*EcoR* I) and 1528-R (*Xho* I) (Table D in S1 Appendix). The resulting DNA fragment was digested with *Xba* I/*BamH* I, then ligated into the pGEX4T-1 vector, generating the plasmid pGEX1528 (Table C in S1 Appendix).

**Co-IP-MS assay**

Each 100 μg protein sample was digested and desalted for the subsequent mass spectrometry analysis (1, 2). The peptide mixtures were analyzed using a nano system (Thermo Scientific, EASY-nLC1200, USA) coupled with a 1,000,000 FWHM high-resolution Nano Orbitrap Fusion Lumos Tribrid Mass Spectrometer system (Thermo Scientific, USA), as previously described (3, 4). Samples, which were introduced with 20 ng/μL bovine serum albumin (BSA) as an internal standard, were loaded onto a nanoViper C18 silica column Acclaim PepMap RSLC (75 μm x 2 cm, 3 μm, 100 Å, Thermo, USA) for trapping, and then were separated on a nanoViper C18 silica column Acclaim PepMap RSLC (75 μm x 25 cm, 2 μm, Thermo, USA). The mobile phase consisted of solvent A (0.1% (v/v) formic acid) and solvent B (80% (v/v) acetonitrile and 0.1% (v/v) formic acid) with a flow rate of 300 nL/min. The gradient program was as follows: 0–4 min, 2% to 10% solvent B; 4–44 min, 10% to 28% B; 44–55 min, 28% to 38% B; 55–60 min, 38% to 55% B; 60–65 min, 55% to 95% B; 65–75 min, 95% B. The eluted peptides were introduced into the mass spectrometer via a nanospray ion source. The Nano Orbitrap Fusion Lumos Tribrid MS, equipped with Ad_van_ced Peak Determination, was operated in a data-dependent acquisition mode with a cycle time of 3 seconds using Xcalibur 4.2.47 software (Thermo Scientific, USA). Full-scan MS spectra (200–2000 m/z) were acquired in the Orbitrap with a resolution of 60,000 at 400 m/z. Precursor ions with an intensity threshold exceeding 4.0 e5 in the quadrupole were selected for MS/MS fragmentation analysis at a normalized collision energy of 3% in the Orbitrap detector with a resolution of 15,000. Dynamic exclusion was set to 25 s to prevent repeated peptide selection. The raw data files from the Orbitrap Lumos were processed using Proteome Discoverer software 2.3 (Thermo Scientific, USA) with the Sequest HT search engine against the proteome sequence databases of the *P. putida* A514 Genome Database (4).

**Supplementary Figures**

**Fig A Validation of OMV purity in *P. putida* A514.** (a) Scanning electron microscopy (SEM) image of isolated OMVs. (b) Western blot analysis of subcellular fractions. β-RNAP was used as a loading control for total cellular lysate; DypB_3152_ served as a control for OMV-resident proteins; and SecY was used as a control for membrane-associated proteins. Their expected localization patterns validate the fractionation integrity. TM: total membrane extracts. Sup: supernatant. Data are representative of two independent biological replicates.

**Fig B Western blot analysis of DypB_2985_ across subcellular fractions in A_2985_ and AR_2985_: whole-cell lysate (Cell), periplasm (Peri), supernatant (Sup), and** **OMVs.** β-RNAP and DsbA were used as the protein expression controls. Data are representative of two independent biological replicates.

**Fig C** **Relative DypB_2985_ levels (a) and enzymatic activity (b) in the periplasm of the relevant mutants.** Relative DypB_2985_ level: the periplasmic DypB_2985_ protein abundance normalized to periplasmic DsbA level. Protein levels were analyzed by Western blot densitometry using ImageJ software. For enzymatic activity measurements, the absorption at 420 nm of periplasmic extracts from strain AR_pUPmin_ was used as a blank control to subtract background activity. DypB_2985_ activity is expressed as units per liter (U/L), where one unit corresponds to the activity in 1 L of culture broth. Data are presented as mean values ± standard deviation of three biological replicates. “***”: *p* value < 0.001 by two-sided Student’s *t*-test.

**Fig D Comparisons of DypB_2985_ protein levels between the mutants and AR_2985_.** DypB_2985_ levels were quantified by Western blot densitometry using ImageJ software. Ratio: DypB_2985_ level in each mutant relative to that in AR_2985_. Data are presented as mean values ± standard deviation of three biological replicates. Cell: the total cellular lysate, Peri: periplasmic fraction, Sup: supernatant fraction. “***”: *p* value < 0.001 by two-sided Student’s *t*-test.

**Fig E Relative periplasmic DypB_2985_ levels in Δ*lpp_1528_* mutants.** DypB_2985_ levels were normalized to periplasmic DsbA as a loading control. Protein levels were quantified by Western blot densitometry using ImageJ software. Data are presented as mean values ± standard deviation of three biological replicates. “***”: *p* value < 0.001 by two-sided Student’s *t*-test.

**Fig F Functional impact of DypB_2985_ truncations on protein expression and stability.** (a-b) ELISA-quantified expression levels of N-terminal (a) and C-terminal (b) truncated DypB_2985_ variants in total lysates. Data are presented as mean values ± standard deviation. n = 6 biological replicates. (c-d) Western blot analysis of protein stability for N-terminal (c) and C-terminal (d) truncations. β-RNAP was used as the loading control. Representative images from two independent biological replicates are shown.

**Fig G DypB_2985_ sequence analysis.** Scatter plot of hydrophobic scores for DypB_2985_ using Expasy-ProtScale. The y-axis represents hydrophobicity score with positive values indicating hydrophobicity and negative values indicating hydrophilicity. The truncated DypB_2985_ amino acid sequences are shown with dashes denoting deleted residues and asterisks (*) denoting stop codons.

**Fig H Interaction of Lpp_1528_ with DypB_2985_ or SecA_4176_ assessed by GST pull-down assay.** Purified His-DypB_2985_ (a), cell lysate from A_2985_ strain (b), and purified His-SecA_4176_ (c) were individually incubated with GST or GST-Lpp_1528_. The initial samples (input) and bound proteins (pull-down fractions) were analyzed by Western blotting using anti-GST or anti-His antibodies, respectively. All blots were independently repeated twice with consistent results.

**Fig I Lpp_1528_ domain structure and localization analysis.** (a) An N-terminal Sec signal peptide (in green) targets Lpp_1528_ to export via the Sec pathway. Lipoprotein signal peptidase (Lsp) recognizes the lipobox (Leu-Ala-Gly-Cys) residues and cleaves the signal peptide. The N-terminal region of the mature lipoprotein contains an outer membrane sorting signal (in blue) at position 2. The C-terminal portion of the polypeptide (in orange) assumes a fold specific function (e.g., hydrolase). (b) Western blot analysis of the Lpp_1528_ protein levels in the whole cellular lysate (Cell), periplasm (Peri), supernatant (Sup), and OMVs in A_2985-1528_, where A_2985_ carries the *lpp_1528_* gene with a N-terminal Myc-tag on the pUCP18 plasmid. Lpp_2985_ was detected with anti-Myc antibody. β-RNAP and DsbA were used as the protein expression controls. Data are representative of two independent biological replicates.

**Fig J DypB_2985_ is exported through the SecYEG translocon.** (a) The gene cluster for Sec components in *P. putida* A514. (b-d) The interaction of SecA_4176_ (b), SecB_5893_ (c), and SecYEG (d) with Lpp_1528_. (e) The interaction between SecYEG and DypB_2985_. The initial cellular lysates (Input) and reserved proteins were detected by Western blot with anti-His and anti-Myc antibodies, respectively. IgG represents the negative control. Data (b-e) are representative of two biological replicates. (f) Investigation of the protein complex formation among DypB_2985_, Lpp_1528_, and SecYEG in AR_2985_. The putative DypB_2985_-containing complexes were separated by Blue Native PAGE and detected by Western blotting using an anti-His antibody specific to the N-terminal His-tag of DypB_2985_. A∆*lpp_1528_* was used as a negative control.

**Fig K OMV protein profiles and growth curves of indicated mutant strains.** (a) SDS-PAGE analysis of OMV protein composition from the indicated mutants. Proteins were separated by SDS-PAGE and stained with Coomassie Brilliant Blue. The gel displays the relative abundance and distribution of OMV-associated proteins across strains. Molecular weight markers (M, in kDa) are shown on the left. (b) Cell growth curves of A_2985_, A∆*lpp_1528_*, and A∆*ompW_4836_* in LB medium.

**Fig** **L Intracellular and periplasmic DypB_2985_ activity in AR_2985_.** DypB_2985_ activity was measured in both the intracellular (Intra) and periplasmic (Peri) fractions of AR_2985_. Strain AR_pUPmin_ was used as a blank control to subtract background activity. Data are presented as mean values ± standard deviation of three biological replicates. “***”: *p* value < 0.001 by two-sided Student’s *t*-test.

**Supplementary Tables**

**Table A Strains used in this study.**

| Strain | Relevant characteristics | Function | Source |
| --- | --- | --- | --- |
| *E. coli* | DH5α | Molecular cloning | Takara |
|  | BL21 (DE3) | Protein expression |  |
| *P. putida* A514 | Wild type | Wild type | (5) |
| ApUPmin | A514 carrying pUCP_min_ | DypB_2985_ localization | (6) |
| A_2985_ | A514 carrying pUHis2985 |  |  |
| A_2985-3152_ | A514 carrying pU2985-3152 | OMV purity assessment | This study |
| A_Flag2985_ | A514 carrying pUFlag2985 | Protein interactions | This study |
| A_2985-4638_ | A514 carrying pU2985-4638 |  |  |
| A_2985-1528_ | A514 carrying pU2985-1528 |  |  |
| A_2985-4836_ | A514 carrying pU2985-4836 |  |  |
| A_2985-1524_ | A514 carrying pU2985-1524 |  |  |
| ARc9n | A514Δ*pyrF*, P_min_::cas9n, P_xylA_::gam-bet-exo | Host strain for genome editing | (1) |
| ARpUPmin | ARc9n carrying pUCP_min_ | Blank reference strain | This study |
| AR_2985_ | ARc9n carrying pUHis2985 | The roles of related proteins in the secretion of DypB_2985_ | This study |
| AΔ*dnaK_4638_* | ARc9n Δ *dnaK_4638_* carrying pUHis2985 |  |  |
| AΔ*lpp_1528_* | ARc9n Δ*lpp_1528_* carrying pUHis2985 |  |  |
| AΔ*ompW_4836_* | ARc9n Δ*ompW_4836_* carrying pUHis2985 |  |  |
| AΔ*duf4938_1524_* | ARc9n Δ*duf4938_1524_* carrying pUHis2985 |  |  |
| AΔ*lpp_1528_*::*lpp_1528_* | ARc9n Δ*lpp_1528_* carrying pUH2985-1528 | Gene complementation | This study |
| AΔ*ompW_4836_*::*opmW_4836_* | ARc9n Δ*opmW_4836_* carrying pUH2985-4836 |  |  |
| A_2985(C6)_ | A514 carrying pU2985(C6) | The recognized C-terminal regions of DypB_2985_ | This study |
| A_2985(C12)_ | A514 carrying pU2985(C12) |  |  |
| A_2985(C18)_ | A514 carrying pU2985(C18) |  |  |
| A_2985(C24)_ | A514 carrying pU2985(C24) |  |  |
| A_2985(N6)_ | A514 carrying pU2985(N6) | The recognized N-terminal regions of DypB_2985_ |  |
| A_2985(N12)_ | A514 carrying pU2985(N12) |  |  |
| A_2985(N18)_ | A514 carrying pU2985(N18) |  |  |
| A_2985(N24)_ | A514 carrying pU2985(N24) |  |  |
| A_2985(C6)-1528_ | A514 carrying pU2985(C6)-1528 | Protein interactions with the truncated DypB_2985_ proteins | This study |
| A_2985(C24)-1528_ | A514 carrying pU2985(C24)-1528 |  |  |
| A_2985(N24)-1528_ | A514 carrying pU2985(N24)-1528 |  |  |
| A_2985(C6)-4836_ | A514 carrying pU2985(C6)-4836 |  |  |
| A_2985(C24)-4836_ | A514 carrying pU2985(C24)-4836 |  |  |
| A_2985(N24)-4836_ | A514 carrying pU2985(N24)-4836 |  |  |
| A_4176-1528_ | A514 carrying pU4176-4836 | Protein interactions in the Sec pathway | This study |
| A_5893-1528_ | A514 carrying pU5893-4836 |  |  |
| A_SecYEG-1528_ | A514 carrying pUSecYEG-4836 |  |  |
| A_SecYEG-2985_ | A514 carrying pUSecYEG-2985 |  |  |
| B_pGEX_ | BL21 carrying pGEX4T-1 | GST pull-down assay | This study |
| B_pGEX1528_ | BL21 carrying pGEX1528 |  |  |
| A_pUHis4176_ | A514 Carrying pUHis4176 |  |  |

**Table B Flag-DypB_2985_ interacted proteins by co-IP-MS assay.**

Data are presented in S1_Table B. xlsx.

**Table C Plasmids used in this study.**

| Plasmid | Relevant characteristics | Function | Source |
| --- | --- | --- | --- |
| pUCP_min_ | pUCP18-Gm derivative, P_min_ promoter | Expression vector | (6) |
| pUHis2985 | pUCP18-Gm derivative, P_min_::His-PputA514_2985 |  |  |
| pU2985-3152 | pUCP18-Gm derivative, P_xylA_::Flag-PputA514_2985, Ph3::Myc- PputA514_3152 | OMV purity assessment | This study |
| pUFlag2985 | pUCP18-Gm derivative, P_xylA_::Flag-PputA514_2985 | coIP assay with DypB_2985_ | This study |
| pU2985-4638 | pUCP18-Gm derivative, P_xylA_::Flag-PputA514_2985, Ph3::Myc- PputA514_4638 |  |  |
| pU2985-1528 | pUCP18-Gm derivative, P_xylA_::Flag-PputA514_2985, Ph3::Myc- PputA514_1528 |  |  |
| pU2985-4836 | pUCP18-Gm derivative, P_xylA_::Flag-PputA514_2985, Ph3::Myc- PputA514_4836 |  |  |
| pU2985-1524 | pUCP18-Gm derivative, P_xylA_::Flag-PputA514_2985, Ph3::Myc- PputA514_1524 |  |  |
| pUCP18-23 | pUCP18 Tet, P_trc_::gRNA(*pyrF*), 0.5 kb *dnaK_4638_* donor up-0.5 kb *dnaK_4638_* donor down, P_min_::*gfp*, P_van_::sgRNA, palindromic sequence | *dnaK_4638_* knockout | This study |
| pUCP18-24 | pUCP18 Tet, P_trc_::gRNA(*pyrF*), 0.5 kb *lpp_1528_* donor up-0.5 kb *lpp_1528_* donor down, P_min_::*gfp*, P_van_::sgRNA, palindromic sequence | *lpp_1528_* knockout |  |
| pUCP18-25 | pUCP18 Tet, P_trc_::gRNA(*pyrF*), 0.5 kb *ompW_4836_* donor up-0.5 kb *ompW_4836_* donor down, P_min_::*gfp*, P_van_::sgRNA, palindromic sequence | *ompW_4836_* knockout |  |
| pUCP18-26 | pUCP18 Tet, P_trc_::gRNA(*pyrF*), 0.5 kb *duf4938_1524_* donor up-0.5 kb *duf4938_1524_* donor down, P_min_::*gfp*, P_van_::sgRNA, palindromic sequence | *duf4938_1524_* knockout |  |
| pUH2985-1528 | pUCP18-Gm derivative, P_min_::PputA514_His-2985, Ph5::PputA514_1528 | *lpp_1528_* complementation | This study |
| pUH2985-4836 | pUCP18-Gm derivative, P_min_::PputA514_His-2985, Ph5::PputA514_4836 | *OmpW_4836_* complementation |  |
| pU2985(C6) | pU2985derivative, P_min_::HisPputA514_2985 (deletion of C-terminal 6 aa) | Protein truncation | This study |
| pU2985(C12) | pU2985derivative, P_min_::HisPputA514_2985 (deletion of C-terminal 12 aa) |  |  |
| pU2985(C18) | pU2985derivative, P_min_::HisPputA514_2985 (deletion of C-terminal 18 aa) |  |  |
| pU2985(C24) | pU2985derivative, P_min_::HisPputA514_2985 (deletion of C-terminal 24 aa) |  |  |
| pU2985(N6) | pU2985derivative, P_min_::HisPputA514_2985 (deletion of N-terminal 6 aa) |  |  |
| pU2985(N12) | pU2985derivative, P_min_::HisPputA514_2985 (deletion of N-terminal 12 aa) |  |  |
| pU2985(N18) | pU2985derivative, P_min_::HisPputA514_2985 (deletion of N-terminal 18 aa) |  |  |
| pU2985(N24) | pU2985derivative, P_min_::HisPputA514_2985 (deletion of N-terminal 24 aa) |  |  |
| pU2985(C6)-1528 | pUCP18-Gm derivative, P_xylA_::Flag-PputA514_2985(C6), Ph3::Myc- PputA514_1528 | coIP assay with the truncated DypB_2985_ proteins | This study |
| pU2985(C24)-1528 | pUCP18-Gm derivative, P_xylA_::Flag-PputA514_2985(C24), Ph3::Myc- PputA514_1528 |  |  |
| pU2985(N24)-1528 | pUCP18-Gm derivative, P_xylA_::Flag-PputA514_2985(N24), Ph3::Myc- PputA514_1528 |  |  |
| pU2985(C6)-4836 | pUCP18-Gm derivative, P_xylA_::Flag-PputA514_2985(C6), Ph3::Myc- PputA514_4836 |  |  |
| pU2985(C24)-4836 | pUCP18-Gm derivative, P_xylA_::Flag-PputA514_2985(C24), Ph3::Myc- PputA514_4836 |  |  |
| pU2985(N24)-4836 | pUCP18-Gm derivative, P_xylA_::Flag-PputA514_2985(N24), Ph3::Myc- PputA514_4836 |  |  |
| pU4176-1528 | pUCP18-Gm derivative, P_xylA_::Flag-PputA514_4176, Ph3::Myc- PputA514_1528 | coIP assay with Lpp_1528_ | This study |
| pU5893-1528 | pUCP18-Gm derivative, P_xylA_::Flag-PputA514_5893, Ph3::Myc- PputA514_1528 |  |  |
| pUSecYEG-1528 | pUCP18-Gm derivative, P_xylA_::Flag-SecYEG, Ph3::Myc-PputA514_1528 | coIP assay with DypB_2985_ | This study |
| pUSecYEG-2985 | pUCP18-Gm derivative, P_xylA_::Flag-SecYEG, Ph3::Myc-PputA514_2985 |  |  |
| pGEX4T-1 | pGEX4T-1 derivative, Amp^r^ | GST pull-down assay | WEIDI |
| PGEX1528 | pGEX4T-1 derivative, P_tac_::GST-PputA514_1528 |  | This study |
| pUHis4176 | pU4176-1528 derivative, P_xylA_::His-PputA514_4176 |  |  |

**Table D Primers used in this study.**

| Primer | Sequence |
| --- | --- |
| Flag (*Xba* I)-F | 5’-CTAGTCTAGAATCCATGGATTACAAGGATGACGACGATAAGGGAG-3’ |
| Flag (*Afl Ⅱ*)-R-O2985 | 5’-ACTGCTTAAGTCCCTTATCGTCGTCATCCTTGTAATCGATCT-3’ |
| 2985 (*Afl Ⅱ*)-F | 5’- GACACTTAAGATGAAGACTTGCCCTGTTCAAGC-3’ |
| 2985 (*Kpn* I)-R | 5’- TCGGGGTACCCATTGAGCTAAGCCGCAGGC-3’ |
| his-2985 R (*Pst* I) | 5’-AAAACTGCAGGTACCCATTGAGCTAAGCCGCAG-3’ |
| his-2985 F (*Xba* I) | 5’-CTAGTCTAGAATGCACCACCACCACCA-3’ |
| 1524 (*Xho* I)-F | 5’-TCTGCCTAGGTCAGTTCGCCATCGACGAACGT-3’ |
| 1524 (*Bln* I)-R | 5’-TACGCTCGAGGTGAATAGCCAACGACCTGTAAACC-3’ |
| 1528 (*Xho* I)-F | 5’-TACGCTCGAGATGAGAATCCTCGGCATTCTTTGC-3’ |
| 1528 (*Bln* I)-R | 5’-TCTGCCTAGGTCATTGCGGGCTCTCTGATGAA-3’ |
| 4638 (*Xho* I)-F | 5’-TACGCTCGAGATGGGCAAAATTATCGGTATCGACC-3’ |
| 4638 (*Bln* I)-R | 5’-TCTGCCTAGGTTACTTGTGGTCTTTGACTTCTTCGAA-3’ |
| 4836 (*Xho* I)-F | 5’-TACGCTCGAGATGAACAAGTCCCTGCTCGGC-3’ |
| 4836 (*Bln* I)-R | 5’-TCTGCCTAGGCTAGAACTTGTAGCCGATACCGACC-3’ |
| P_trc_-F (*Pst* I) | 5’-CAAACTGCAGATGTGAGTTAGCGCGAATTGAT-3’ |
| P_trc_ (1528)-R | 5’-GGGTTTCGGGCGCCTTGTCGTGTGTGAAATTGTTATCCGCTCAC-3’ |
| Scaffold (1528)-F | 5’-CGACAAGGCGCCCGAAACCCGTTTTAGAGCTAGAAATAGCAAGTT-3’ |
| rrnbT7-R | 5’-GCCGGAATTCAAGCTTCATGGCCACGCAGTCCAGAGAAATCGGCTAG-3’ |
| 1528 UD-F (*Xba* I） | 5’-TAGCTCTAGATTTGCGCTCATCGGTGTTGTCC-3’ |
| 1528 UD-R (P*pyrF*） | 5’-CGGTGATCGCCGAAGACAAGCTGATCCTCACCGGTATCGGCA-3’ |
| *pyrF*-F | 5’-CTTGTCTTCGGCGATCACCG-3’ |
| *pyrF*-R | 5’-TCAGGCGATTTCGGCAACC-3’ |
| 1528 DD-F (*pyrF*) | 5’-GGTTGCCGAAATCGCCTGAATTTGGCCGCGATCTGCAG-3’ |
| 1528 DD-R (*Sac* I) | 5’-TGTCGAGCTCTTGAAATGGCCGAAAGCTACGGC-3’ |
| P_trc_ (1524)-R | 5’-GCGCAGCCCGCCAGTACGAATGTGTGAAATTGTTATCCGCTCAC-3’ |
| Scaffold (1524)-F | 5’-TTCGTACTGGCGGGCTGCGCGTTTTAGAGCTAGAAATAGCAAGTT-3’ |
| 1524 UD-F (*Xba* I) | 5’-TAGCTCTAGAGCCATCGCGGGCTTATTGAGG-3’ |
| 1524 UD-R (P*pyrF*) | 5’-CGGTGATCGCCGAAGACAAGCCATTTCGTTGGTCAGCGGTCG-3’ |
| 1524 DD-F (*pyrF*) | 5’-GGTTGCCGAAATCGCCTGAGCAGTGCACCCGATATCGAATTGAAA-3’ |
| 1524 DD-R (*Sac* I) | 5’-TGTCGAGCTCCGCCCTGTTGAGCCAGTGC-3’ |
| P_trc_ (4836)-R | 5’-GGTTGGTGCCCGCCAAGGGGTGTGTGAAATTGTTATCCGCTCAC-3’ |
| Scaffold (4836)-F | 5’-CCCCTTGGCGGGCACCAACCGTTTTAGAGCTAGAAATAGCAAGTT-3’ |
| 4836 UD-F (*Xba* I) | 5’-TAGCTCTAGATTATGAGGAAGGCTTCGATGCGC-3’ |
| 4836 UD-R (P*pyrF*) | 5’-CGGTGATCGCCGAAGACAAGCCGTTCGATCCTTTGACTCAGCTC-3’ |
| 4836 DD-F (*pyrF*) | 5’-GGTTGCCGAAATCGCCTGAATCCCATCCAAGACCCTGTTTTTACAG-3’ |
| 4836 DD-R (*Sac* I) | 5’-TGTCGAGCTCGCGCACCACCGAAAGTATCGTC-3’ |
| P_trc_ (4638)-R | 5’-GGGTCACGGTGGGCAGCATCTGTGTGAAATTGTTATCCGCTCAC-3’ |
| Scaffold (4638) -F | 5’-GATGCTGCCCACCGTGACCCGTTTTAGAGCTAGAAATAGCAAGTT-3’ |
| 4638 UD-F (*Xba* I) | 5’-TAGCTCTAGAGCAAGACAGCGACGACCGTG-3’ |
| 4638 UD-R (P*pyrF*) | 5’-CGGTGATCGCCGAAGACAAGTGGTCAAGGCCTTAACCGGATCG-3’ |
| 4638 DD-F (*pyrF*) | 5’-GGTTGCCGAAATCGCCTGACTCCACATTCCTACACAAAGCGTTTATC-3’ |
| 4638 DD-R (*Sac* I) | 5’-TGTCGAGCTCGCCTTCAGCATTGCCCAGGTAG-3’ |
| 2985-F (*Pst* I) | 5’-TGCACTGCAGATGAAGACTTGCCCTGTTCAAGCC-3’ |
| 2985(N6)-F (*Pst* I) | 5’-TGCACTGCAGATGGCCCCTTTAGAGCCGCA-3’ |
| 2985(N12)-F (*Pst* I) | 5’-TGCACTGCAGATGTCCGTCTGCCATCCCATCAC-3’ |
| 2985(N18)-F (*Pst* I) | 5’-TGCACTGCAGATGACCCGCAGCGCGATCT-3’ |
| 2985(N24)-F (*Pst* I) | 5’-TGCACTGCAGATGATGGTTGCTACCGTTACCCC-3’ |
| 2985-R (*Kpn* I) | 5’-TATAGGTACCCTAGTGGTGGTGGTGGTGGTGAGCCGCAGGCGGGCG-3’ |
| 2985-R(C6) (*Kpn* I) | 5’-TATAGGTACCCTAGTGGTGGTGGTGGTGGTGGTCGGCCAGCGCG-3’ |
| 2985-R(C12) (*Kpn* I) | 5’-TATAGGTACCCTAGTGGTGGTGGTGGTGGTGGTCGGCCGAAGGCACG-3’ |
| 2985-R(C18) (*Kpn* I) | 5’-TATAGGTACCCTAGTGGTGGTGGTGGTGGTGAAACAGGCCGCCGGTC-3’ |
| 2985-R(C24) (*Kpn* I) | 5’-TATAGGTACCCTAGTGGTGGTGGTGGTGGTGCGCCGTGCTGAAGTCCA-3’ |
| 4176-F (*Spe* I) | 5’-GACAACTAGTATGTTTGCGCCTTTGTTAAAGAAACT-3’ |
| 4176-R (*BamH* I) | 5’-CATAGGATCCAGTTAGTTGATTTCGCCGTGGCAG-3’ |
| 5893-F (*Spe* I) | 5’-GACAACTAGTATGACTGACCAACAGAACACCGA-3’ |
| 5893-R (*BamH* I) | 5’-CATAGGATCCAGTTATTGAACAGTCGACGAACCTTCTG-3’ |
| 1528-F (*EcoR* I) | 5’-GTCAGAATTCATGAGAATCCTCGGCATTCTTTGC-3’ |
| 1528-R (*Xho* I) | 5’-GACTCTCGAGTCATTGCGGGCTCTCTGATGA-3’ |

**References**

1.	Liang C, Lin L, Wang X, Zhu W. The secretion of *Pseudomonas* unconventional peroxidase facilitates extracellular carbon acquisition from heterogeneous lignin. Commun Biol. 2025;8(1):1318.

2.	Winiewski JR, Zougman A, Nagaraj N, Mann M. Universal sample preparation method for proteome analysis. Nat Methods. 2009;6(5):359-62.

3.	Espadas G, Borràs E, Chiva C, Sabidó E. Evaluation of different peptide fragmentation types and mass analyzers in data-dependent methods using an Orbitrap Fusion Lumos Tribrid mass spectrometer. Proteomics. 2017;17(9):1600416.

4.	Liang C, Lin L, Xu T, Kang G, Liu ZH, Li BZ. The *Pseudomonas* ligninolytic catalytic network reveals the importance of auxiliary enzymes in lignin biocatalysts. PNAS. 2025;122(4):e2417343122.

5.	Lin L, Cheng Y, Pu Y, Sun S, Li X, Jin M, et al. Systems biology-guided biodesign of consolidated lignin conversion. Green Chem. 2016;18(20):5536-47.

6.	Lin L, Wang X, Cao LH, Xu M. Lignin catabolic pathways reveal unique characteristics of dye‐decolorizing peroxidases in *Pseudomonas putida*. Environ Microbiol. 2019;21:1847–63.
